# Dietary Reactive Oxygen Species and Oxidative Stress: Pathogenic Mechanisms Linking Food and Feed Exposure to Disease in Humans and Companion Animals

**DOI:** 10.3390/biology15171519

**Published:** 2026-09-03

**Authors:** Hye-Jin Park, Hyo-Min Kim

**Affiliations:** Department of Veterinary Medicine, College of Veterinary Medicine, Konkuk University, Seoul 05029, Republic of Korea

**Keywords:** reactive oxygen species (ROS), oxidative stress, dietary pro-oxidants, ROS-generating factors in unprocessed foods and feeds, ROS-generating factors in processed foods and feeds, human, companion animals, comparative pathology

## Abstract

Oxidative stress, arising from an imbalance between reactive oxygen species (ROS) and antioxidant defenses, is a shared pathogenic mechanism underlying numerous chronic diseases in humans and, increasingly, in companion animals that consume structurally similar processed diets. This narrative review synthesizes current evidence on dietary and feed-derived sources of ROS, spanning intrinsic pro-oxidant systems in unprocessed foods (lipid hydroperoxides, heme iron, and polyphenol autoxidation) and heat processing-generated toxicants (advanced glycation end products, acrylamide, heterocyclic aromatic amines, and lipid oxidation products), and examines the molecular mechanisms that link these compounds to organ-specific and systemic disease, including receptor-mediated signaling, cytochrome P450 activation, mitochondrial dysfunction, and antioxidant depletion. Comparative analysis across humans, dogs, and cats reveals parallel disease burdens in metabolic syndrome, chronic kidney disease, inflammatory bowel disease, and cancer, alongside instructive species-specific divergences in atherosclerosis and neurodegenerative disease driven by differences in lipoprotein metabolism and toxicokinetics. The review further reassesses canonical dietary antioxidants, including polyphenols and omega-3 polyunsaturated fatty acids, as context-dependent redox modulators capable of both cytoprotective and pro-oxidant activity. Collectively, these findings position dietary and feed processing as an under-recognized, modifiable contributor to chronic disease across species. In this context, companion animals emerge as valuable naturalistic translational models for studying diet-induced oxidative pathology. Priority research needs include standardized dose–response and biomarker frameworks, characterization of gut microbiome-mediated effects, and assessment of combinatorial pro-oxidant exposures.

## Simple Summary

Every day, the food we eat and the food we feed our pets can contain small amounts of natural chemicals that create so-called “reactive oxygen molecules” inside the body. In small amounts, these molecules are harmless and even useful, but too many of them damage cells and contribute to diseases such as diabetes, heart disease, kidney disease, and cancer. This problem is not limited to people: dogs and cats that eat commercially processed pet food face remarkably similar chemical exposures and develop many of the same diseases. This review brings together current scientific knowledge about which foods and which cooking or manufacturing processes generate these harmful molecules, how they damage the body, and which diseases they are linked to in both humans and companion animals. It also compares how differently the human and animal body handles these substances and examines certain “healthy” dietary compounds, such as plant polyphenols, that can either protect against or, depending on the amount consumed, contribute to this damage. By connecting research on humans, dogs, and cats living in the same households, this review highlights shared health risks and points to future research that could improve dietary guidance and disease prevention for both people and their pets.

## 1. Introduction

### 1.1. Reactive Oxygen Species Overview

Reactive oxygen species (ROS) are chemically reactive molecules derived from molecular oxygen, encompassing free radicals such as the superoxide anion (O_2_•^−^), hydroxyl radical (•OH), and peroxyl radicals (ROO•), as well as non-radical oxidants including hydrogen peroxide (H_2_O_2_), singlet oxygen (^1^O_2_), and hypochlorous acid (HOCl) [1,2]. Under physiological conditions, ROS are continuously generated as by-products of aerobic metabolism, predominantly through mitochondrial electron transport-chain activity, NADPH oxidase (NOX) complexes, xanthine oxidase, and cytochrome P450 enzymes [3,4]. At low to moderate concentrations, ROS serve as essential second messengers in redox signaling pathways, regulating cell proliferation, differentiation, apoptosis, immune defense, and vascular homeostasis [5,6]. This beneficial mode of ROS action is termed oxidative eustress, as distinct from oxidative distress, the damaging state that arises when ROS overwhelm antioxidant defenses.

Cellular antioxidant defense systems maintain redox homeostasis through enzymatic mechanisms such as superoxide dismutase (SOD), catalase (CAT), glutathione peroxidase (GPx), and thioredoxin reductase, as well as non-enzymatic antioxidants, including glutathione (GSH), vitamins C and E, carotenoids, and polyphenols [7,8]. When the production of ROS exceeds the capacity of these antioxidant defenses, a state of oxidative stress emerges. This imbalance leads to indiscriminate oxidative damage to biomolecules, including lipid peroxidation of membrane phospholipids, oxidative modification of proteins (carbonylation, nitrosylation), and DNA strand breaks and base modifications (8-hydroxy-2′-deoxyguanosine, 8-OHdG), collectively impairing cellular function and viability [9,10].

Chronic oxidative stress has been implicated in the pathogenesis of a broad spectrum of diseases, including cardiovascular disease, type 2 diabetes mellitus (T2DM)**,** neurodegenerative disorders (Alzheimer’s and Parkinson’s diseases), chronic kidney disease, and various cancers [10,11,12,13]. Accumulating evidence further indicates that oxidative stress is a critical driver of chronic low-grade inflammation, including a shared pathological denominator across metabolic and age-related diseases through activation of redox-sensitive transcription factors such as nuclear factor kappa B (NF-κB) and nuclear factor erythroid 2-related factor 2 (Nrf2) [14,15].

### 1.2. Diet-Induced Oxidative Stress

Diet represents one of the most significant and modifiable environmental determinants of systemic oxidative stress [16]. Dietary components can modulate ROS production and antioxidant capacity through multiple mechanisms, thereby tipping the redox balance toward either oxidative damage or cytoprotection. Postprandial oxidative stress such as a transient increase in circulating oxidative markers following a meal is now recognized as a physiologically relevant phenomenon that, when chronically sustained, contributes to endothelial dysfunction, insulin resistance, and cardiometabolic disease [17,18].

Diets rich in saturated fatty acids and trans fats promote mitochondrial dysfunction and activate NOX enzymes, increasing superoxide generation [19]. Excessive intake of carbohydrates, particularly refined sugars, stimulates glycolysis and the hexosamine pathway, augmenting advanced glycation end-product (AGE) formation and receptor for AGE (RAGE)-mediated ROS production [20,21]. High dietary iron and copper can catalyze the Fenton reaction, generating highly reactive hydroxyl radicals [22]. Conversely, habitual consumption of antioxidant-rich foods such as fruits, vegetables, whole grains, and legumes replenishes endogenous antioxidant pools, upregulates Nrf2-mediated gene expression, and attenuates markers of oxidative stress [23,24].

Of particular relevance are exogenous ROS and pro-oxidant compounds directly introduced through food. Thermally processed foods contain substantial quantities of lipid oxidation products (LOPs), including aldehydes (4-hydroxynonenal and malondialdehyde), acrolein, and oxidized fatty acids, which are absorbed from the gastrointestinal tract and exert systemic pro-oxidant effects [25,26]. Additionally, the gut microbiome plays an emerging role in mediating diet-induced oxidative stress, as dietary patterns shape microbial community composition, which, in turn, influences intestinal-barrier integrity, endotoxemia-driven oxidative signaling, and systemic redox balance [27,28].

### 1.3. Increasing Global Consumption of Processed Foods

Over recent decades, global dietary patterns have undergone a profound transition characterized by the displacement of minimally processed whole foods by ultra-processed food products (UPFPs) [29]. The NOVA classification system classifies all foods and food products into four groups according to the extent and purpose of industrial processing: unprocessed or minimally processed foods, processed culinary ingredients, processed foods, and ultra-processed foods [30]. Within this system, ultra-processed foods are defined as industrial formulations manufactured from substances extracted from foods or synthesized from food constituents, typically containing little, if any, whole food and incorporating numerous additives to enhance palatability, texture, and shelf life [31]. Current estimates suggest that UPFPs account for 25–60% of total daily energy intake in high-income countries, with similar trends emerging across middle-income nations, driven by urbanization, economic development, and globalization of food supply chains [32,33].

Ultra-processed foods are characteristically high in refined sugars, sodium, saturated and trans fats, and synthetic additives (artificial colorants, emulsifiers, and preservatives) while being depleted of dietary fiber, micronutrients, and phytochemicals [34,35]. Industrial food processing operations, including high-temperature extrusion, frying, baking, and spray-drying, generate a range of pro-oxidant compounds through Maillard reactions, lipid peroxidation, and acrylamide formation [36,37]. Epidemiological studies have robustly associated higher UPFP consumption with elevated biomarkers of oxidative stress and inflammation, as well as increased risk of obesity, T2DM, cardiovascular disease, colorectal cancer, and all-cause mortality [38,39].

Mechanistically, the pro-oxidant effects of UPFPs likely arise from a synergistic combination of direct oxidant delivery, disruption of antioxidant micronutrient supply, promotion of endogenous ROS-generating metabolic pathways, and adverse remodeling of the gut microbiota [40,41]. The concurrent depletion of dietary antioxidants—most notably, vitamins C and E, selenium, zinc, and polyphenols—further impairs antioxidant defenses and amplifies susceptibility to oxidative damage [42]. These converging mechanisms collectively establish a compelling biological basis for the epidemiological associations between processed food consumption and chronic disease burden.

### 1.4. Processed Feed Consumption in Companion Animals

Contemporary companion animals—predominantly domestic dogs (*Canis lupus familiaris*) and cats (*Felis catus*)—subsist almost exclusively on commercially manufactured, processed pet foods, a dietary paradigm that has become firmly established over the latter half of the twentieth century [43,44]. The global pet food market exceeded USD 150 billion in 2024 and continues to expand, reflecting the deepening integration of companion animals into human family structures and the corresponding humanization of pet care [45]. Commercially produced dry kibble and semi-moist extruded products now constitute the primary caloric source for the majority of companion animals in developed countries, with wet canned foods, treats, and a growing segment of raw and minimally processed diets comprising the remainder [46].

Commercial pet food manufacturing employs thermal processing methods, including principally high-temperature, high-pressure extrusion for dry kibble production, that are broadly analogous to those used in human food processing [47,48]. These procedures are known to generate significant quantities of Maillard reaction products, advanced glycation end products (AGEs), oxidized lipids, and acrylamide, compounds with established pro-oxidant and cytotoxic properties [49,50]. Notably, the high fat content of many pet food formulations—particularly those incorporating rendered animal by-products—renders them especially susceptible to lipid oxidation during manufacturing, storage, and post-opening exposure [51,52].

Despite the dominant role of processed feeds in companion-animal nutrition, the relationship between dietary ROS exposure and oxidative stress-related disease in these species remains underexplored relative to the human literature [53]. Dogs and cats are afflicted by a strikingly parallel spectrum of chronic diseases relative to those seen in humans—including obesity, T2DM, cardiovascular disease, chronic kidney disease, inflammatory bowel disease, and cancer—many of which share oxidative stress as a central pathophysiological mechanism [54,55]. Given that companion animals share domestic environments with their owners and are exposed to structurally similar dietary processing conditions, they represent both a potentially vulnerable population and a uniquely relevant translational model for investigating diet-induced oxidative pathology [56,57].

The present review synthesizes current evidence concerning the dietary sources and biochemical identity of exogenous ROS and pro-oxidant compounds in human and companion-animal diets, delineates the pathogenic mechanisms through which dietary oxidative stress contributes to organ-specific and systemic disease, and evaluates current and emerging dietary strategies to mitigate oxidative stress burden. By integrating perspectives from human nutrition science, veterinary medicine, and food chemistry, this review aims to provide a comprehensive framework for understanding diet-induced oxidative pathology across species and to identify opportunities for translational research that may benefit both human and companion-animal health.

## 2. Sources of Dietary ROS and ROS-Inducing Compounds

### 2.1. Direct ROS-Generating Factors in Unprocessed Foods

**Figure 1:**
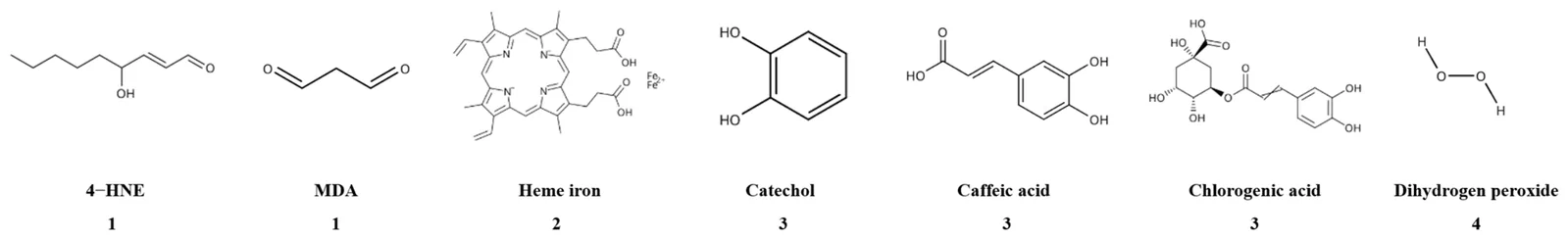
Potential dietary ROS-generating compounds in unprocessed foods. Numbers correspond to the compound/factor categories listed in Table 1. Abbreviations are as follows: 4-HNE, 4-hydroxy-2-nonenal; MDA, malondialdehyde.

Unprocessed or minimally handled foods harbor intrinsic chemical and enzymatic pro-oxidant systems that generate or deliver biologically active oxidants directly to the gastrointestinal tract and systemic circulation. Chief among these are pre-formed lipid hydroperoxides (LOOHs), which accumulate during post-slaughter handling and storage of lipid-rich foods, and heme iron from red meat and fish, which catalyzes Fenton-type hydroxyl radical generation independent of thermal treatment. Plant-derived foods contribute additional factors: polyphenols, conventionally regarded as antioxidants, can generate superoxide and hydrogen peroxide via quinone autoxidation under specific conditions; ascorbic acid co-occurring with redox-active transition metals drives a cyclic Fenton amplification; and enzymatic systems in fresh milk and honey produce H_2_O_2_ directly. Together, these factors impose a baseline dietary pro-oxidant burden that precedes and compounds the additional ROS load from thermal processing, with the magnitude determined by dietary composition, antioxidant co-ingestion, and species-specific gastrointestinal antioxidant defenses (Figure 1 and Table 1).

#### 2.1.1. Oxidized Lipids and Lipid Hydroperoxides: Autoxidation, Enzymatic Pathways, and Systemic Bioavailability

In unprocessed foods, lipid peroxidation proceeds as an Fe^2+^-catalyzed radical chain that yields LOOH as the principal stable product, together with reactive secondary aldehydes such as 4-hydroxynonenal (4-HNE) and malondialdehyde (MDA) (Figure 1, Table 1) [58,59]. Raw muscle foods and lipid-rich plant ingredients carry active enzymatic pro-oxidant systems that pre-load these foods with oxidants before any thermal processing [58,59,60]. Lipoxygenase in fish, legumes, and plant-derived feed ingredients dioxygenates polyunsaturated fatty acids (PUFAs) without the autoxidation lag phase, accelerating oxidative deterioration of fresh fish and raw pet-food ingredients [61,62]. In post-mortem fish muscle, ATP catabolism converts xanthine dehydrogenase to xanthine oxidase, a second enzymatic source of superoxide and hydrogen peroxide that sustains Fenton chemistry as rigor-mortis iron is released [58,60,63].

#### 2.1.2. Pre-Formed H_2_O_2_ in Unprocessed Foods: Enzymatic and Autoxidative Origins

Hydrogen peroxide is not only an intracellular metabolite but a pre-formed, measurable constituent of several unprocessed foods, generated enzymatically and autoxidatively within the food matrix (Table 1) [64,65]. Its biological significance depends on its fate in the gastrointestinal tract: gastric catalase and mucosal peroxidases clear a substantial fraction, but once food H_2_O_2_ exceeds this scavenging capacity or co-ingested redox-active metals drive its Fenton conversion to •OH^−^, the oxidant reaches the intestinal epithelium in reactive form [66,67], favoring mucosal oxidative stress and epithelial DNA damage [67,68]. This convergence is especially relevant to dogs on raw meat-and-bone diets, where milk-derived xanthine oxidase, heme iron, and polyphenol autoxidation products may coincide to create an acute gastric pro-oxidant milieu warranting consideration in dietary risk assessment.

#### 2.1.3. Heme Iron as a Dietary Fenton Catalyst

Red meat and fish are the principal dietary sources of heme iron, supplied as oxymyoglobin, deoxymyoglobin, oxyhemoglobin, and cytochrome-associated iron in muscle tissue [69,70]. Under physiological conditions, the porphyrin ring of intact heme proteins largely shields Fe^2+^ from H_2_O_2_, limiting Fenton-type chemistry; however, gastric acid and proteolysis partially denature ingested heme proteins, releasing the ferrous iron center to catalyze Fe^2+^ + H_2_O_2_ → Fe^3+^ + •OH + OH^−^ [71]. The resulting hydroxyl radicals initiate lipid peroxidation of co-ingested PUFA in the gastric lumen, generating reactive aldehydes such as MDA and 4-HNE that are absorbed and distributed systemically, while free Fe^2+^ additionally accelerates chain propagation by catalyzing homolytic decomposition of pre-existing LOOH [69,70]. Epidemiological data from Mediterranean cohorts indicate that heme iron intake from meat and fish is independently and positively associated with plasma thiobarbituric acid-reactive substance (TBARS) concentrations, whereas non-heme iron intake shows no such association, underscoring the specific pro-oxidant activity of the heme moiety itself rather than elemental iron [72]. In vitro studies in human colonocyte models confirm that hemin generates intracellular ROS and 8-oxo-deoxyguanosine at luminal-relevant concentrations, whereas equimolar inorganic iron salts do not, reinforcing the unique catalytic role of the porphyrin–iron complex [73]. This pro-oxidant pathway is amplified in companion animals consuming high-meat diets, particularly cats, whose obligate carnivory necessitates high animal protein intake and whose diminished Phase II conjugation capacity may impair detoxification of heme-derived lipid peroxidation products [74,75].

#### 2.1.4. Polyphenol Autoxidation and Quinone-Mediated ROS Generation

Polyphenols—the most abundant phytochemicals in fruits, vegetables, legumes, and beverages such as tea and coffee—are conventionally regarded as dietary antioxidants yet possess an inherent pro-oxidant capacity under physiologically relevant conditions [67,68]. The catechol moieties of flavonoids and hydroxycinnamic acids are oxidized by polyphenol oxidase (PPO), a copper-containing enzyme widespread in raw plant tissue, to electrophilic o-quinones; the resulting autoxidation cascade proceeds through sequential one-electron steps that generate superoxide and, following dismutation, H_2_O_2_ as long as oxygen and substrate remain available, constituting a catalytic cycle of ROS generation particularly active in mechanically disrupted tissues such as freshly cut fruit [64,67,76,77]. The generated o-quinones are, themselves, reactive electrophiles that oxidize protein thiols, ascorbate, and glutathione, depleting cellular antioxidant pools and amplifying net pro-oxidant burden [77]. This effect is dose- and context-dependent: at low concentrations, polyphenol-derived H_2_O_2_ functions as a redox signal activating the Nrf2/antioxidant response element (ARE) antioxidant gene battery, whereas at higher concentrations or with co-present redox-active metals, cytotoxic outcomes including protein carbonylation and DNA strand breakage predominate [64,68]. In companion-animal nutrition, raw diets containing high loads of plant-derived ingredients with active PPO may contribute a polyphenol-driven oxidant flux that interacts additively with LOOH^−^ and heme iron-derived ROS [78,79].

#### 2.1.5. Ascorbic Acid and Transition-Metal Synergy: Ascorbate-Driven Cyclic Fenton Amplification

Ascorbic acid (vitamin C) is among the most potent water-soluble antioxidants in the human diet yet harbors a conditional pro-oxidant capacity that becomes operative when redox-active transition metals are available in catalytically competent form [80,81]. Ascorbate reduces Fe^3+^ to Fe^2+^, which immediately enters the Fenton reaction, while the resulting Fe^3+^ is rapidly re-reduced by additional ascorbate, sustaining •OH production in a continuous cycle that proceeds substantially faster than Fe^2+^-only Fenton chemistry; an analogous cycle operates with copper, and in vivo co-administration of ascorbic acid and Cu^2+^ in mice produces systemic protein oxidation and renal tubular injury, confirming pathophysiological relevance [82,83,84]. Under normal dietary conditions, sequestration of labile iron and copper into ferritin, transferrin, ceruloplasmin, and metallothionein largely prevents this ascorbate-driven Fenton chemistry at physiological vitamin C intakes; however, this protection can be overcome by mucosal iron loading—such as that which follows high-heme-iron meals—creating a luminal environment in which co-ingested ascorbate from plant foods paradoxically amplifies •OH production rather than providing antioxidant protection [81,82]. Thus, the net physiological effect of dietary ascorbate on redox balance depends critically on the labile metal pool available in the gut lumen at the time of ingestion, which is, itself, determined by meal composition and species-specific gastrointestinal physiology [80].

**Table 1:** Dietary ROS-generating factors in unprocessed human foods and companion-animal feeds: sources, ROS mechanisms, biomarkers, and species relevance (Section 2.1).

Section	Compound/Factor	Chemical Class	Dietary Source (Human)	Dietary Source (Pet Feed)	Primary ROS-Generating Mechanism	Key Oxidative Biomarkers	Species Relevance & Key References
**2.1. Direct ROS-Generating Factors in Unprocessed Foods**							
2.1	Lipid hydroperoxides (LOOH) & secondary LOPs (4-HNE and MDA) (**1**)	Primary & secondary lipid oxidation products	Red meat, fatty fish, and raw dairy; accumulate during post-slaughter handling and cold storage	Raw/minimally processed meat ingredients; rendered animal fats susceptible to oxidation during storage and post opening	LOOH decomposition (Fenton) → •OH; 4-HNE Michael adducts on Complex I (NDUFV1) → ETC electron leakage → mitochondrial O_2_•^−^; 4-HNE inactivates thioredoxin reductase → impaired Prx peroxide clearance	Plasma MDA/TBARS ↑; urinary 8-OHdG ↑; 8-isoprostane ↑; 4-HNE-protein adducts (Western blot/MS)	[85,86,87,88,89,90,91,92,93,94,95]
2.1	Heme iron (myoglobin/hemoglobin Fe^2+^) (**2**)	Hemoprotein-bound transition metal (non-thermal)	Red and processed meats and fish (independent of thermal treatment)	Meat by-products and blood meal in wet and dry pet food	Fe^2+^ + H_2_O_2_ → Fe^3+^ + •OH (Fenton); catalyzes LOOH decomposition → alkoxyl & peroxyl radicals; accelerates colonic lipid peroxidation	Plasma •OH adducts; colon mucosal MDA ↑; 8-OHdG ↑ in colonic epithelium	[73,96,97,98,99,100,101]
2.1	Pro-oxidant polyphenols (quinone autoxidation) (**3**)	Polyphenolic secondary metabolites (context-dependent)	Tea, coffee, wine, certain fruits and vegetables (high concentration or co-ingestion with redox-active metals)	Plant-based or ‘grain-free’ pet food ingredients (limited data)	Polyphenol → quinone → semiquinone → O_2_•^−^ & H_2_O_2_ via autoxidation; ascorbate-driven cyclic Fenton amplification with co-present Cu^2+^/Fe^2+^	Intracellular ROS (DCFH-DA); GSH depletion in vitro; context-dependent in vivo	[102,103,104,105,106,107,108]
2.1	Enzymatic H_2_O_2_ (xanthine oxidase and glucose oxidase) (**4**)	Enzymatic oxidant system in fresh foods	Raw milk and honey (enzymatic H_2_O_2_ production as metabolic by-product)	Milk-based treats and honey-containing formulations	Enzymatic H_2_O_2_ generation; catalytic Fenton in presence of Fe^2+^ → •OH; direct mucosal oxidant	H_2_O_2_ concentration in food matrix; indirect mucosal ROS burden	[109,110,111,112,113,114,115,116,117,118]

Abbreviations: ROS, reactive oxygen species; LOOH, lipid hydroperoxide; LOPs, lipid oxidation products; 4-HNE, 4-hydroxy-2-nonenal; MDA, malondialdehyde; Fe^2+^, ferrous iron; Fe^3+^, ferric iron; H_2_O_2_, hydrogen peroxide; •OH, hydroxyl radical; O_2_•^−^, superoxide anion radical; NDUFV1, NADH:ubiquinone oxidoreductase core subunit V1; ETC, electron transport chain; Prx, peroxiredoxin; TBARS, thiobarbituric acid-reactive substances; 8-OHdG, 8-hydroxy-2′-deoxyguanosine; MS, mass spectrometry; Cu^2+^, cupric ion; DCFH-DA, 2′,7′-dichlorodihydrofluorescein diacetate; GSH, reduced glutathione. → indicates mechanistic relationships; ↑ indicates an increase.

### 2.2. Processing-Induced ROS-Generating Compounds

**Figure 2:**
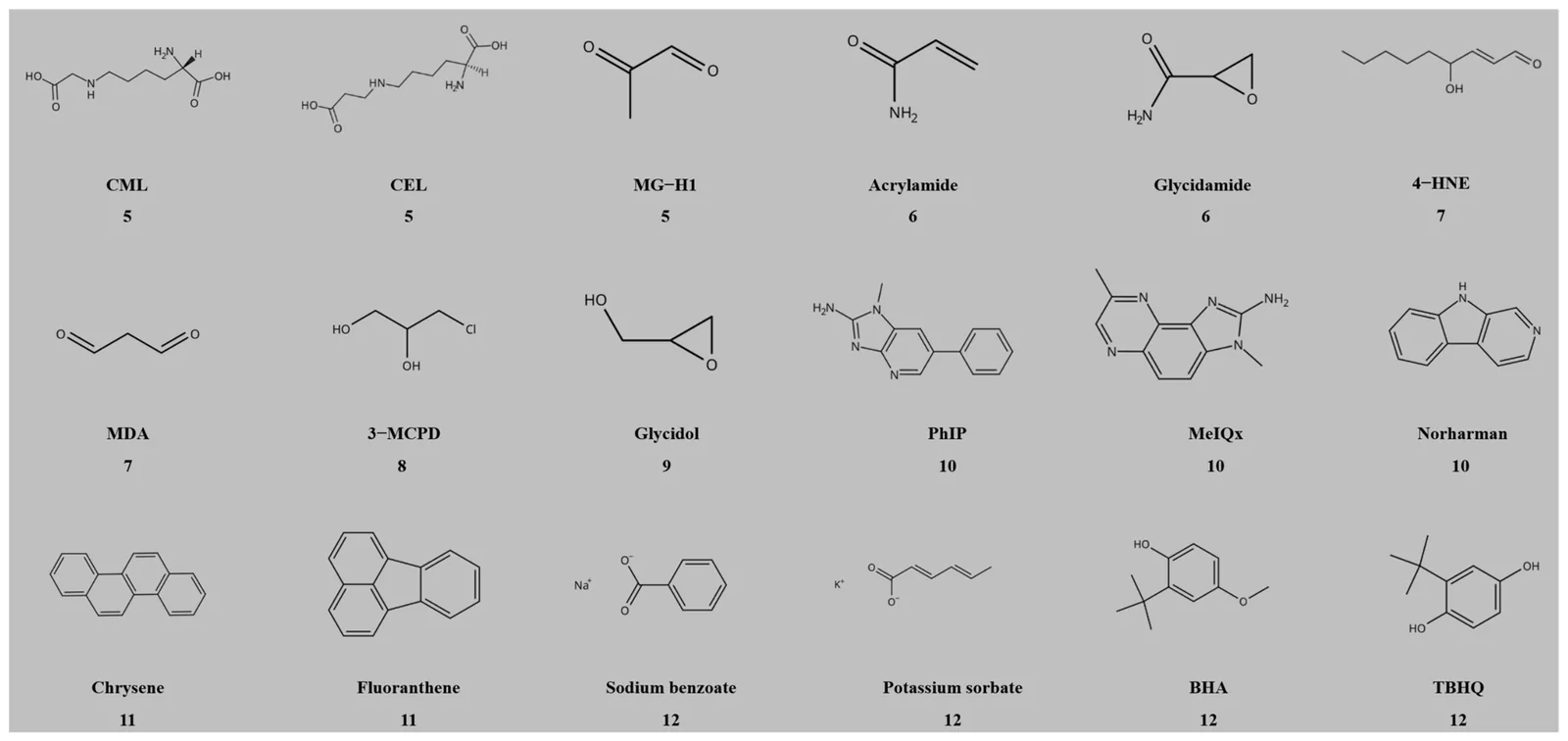
Representative processing-induced pro-oxidant compounds. Numbers correspond to the compound/factor categories listed in Table 2. Abbreviations are as follows: CML, Nε-carboxymethyllysine; CEL, Nε-carboxyethyllysine; MG-H1, methylglyoxal-derived hydroimidazolone 1; 4-HNE, 4-hydroxy-2-nonenal; MDA, malondialdehyde; 3-MCPD, 3-monochloropropane-1,2-diol; PhIP, 2-amino-1-methyl-6-phenylimidazo [4,5-b]pyridine; MeIQx, 2-amino-3,8-dimethylimidazo [4,5-f]quinoxaline; BHA, butylated hydroxyanisole; TBHQ, tert-butylhydroquinone.

Thermal and technological processing of foods and feeds not only modifies nutrient content but also generates a broad spectrum of processing-induced contaminants (PICs) that disrupt cellular redox homeostasis. Despite their structural diversity, these neo-formed molecules share a unifying mechanistic thread: upon absorption and metabolic activation, they converge on ROS generation through NADPH oxidase activation, mitochondrial electron transport impairment, redox cycling, and depletion of endogenous antioxidant defenses [119,120] (Figure 2). This convergence is particularly relevant to chronically processed diets in both humans and companion animals, where cumulative exposure to multiple PICs may produce additive or synergistic pro-oxidant burdens. The following subsections address the major PIC classes—AGEs; acrylamide; lipid oxidation products, including chloropropanols and glycidyl esters; heterocyclic aromatic amines (HCAs); polycyclic aromatic hydrocarbons (PAHs); N-nitroso compounds; and preservative-associated toxicants—and their mechanistic links to ROS generation across species [121,122] (Figure 2 and Table 2).

#### 2.2.1. AGEs and ROS Signaling

AGEs are generated during the Maillard reaction, in which reducing sugars non-enzymatically condense with amino groups of proteins, peptides, or free amino acids under thermal processing. High levels accumulate in heat-processed protein- and fat-rich foods such as roasted meats and baked products; dietary AGE bioavailability is estimated at ~10%, with absorbed fractions accumulating in tissues with age and metabolic disease [123]. Beyond a receptor-independent pro-oxidant effect in which AGE-modified proteins chelate transition metals to facilitate Fenton-type •OH generation, AGEs also signal through the receptor for advanced glycation end products (RAGE) on endothelial, immune, and parenchymal cells, activating membrane-associated NADPH oxidase (particularly Nox1/Nox2) to enhance superoxide and downstream H_2_O_2_ production [124,125]. In vascular smooth muscle and mesangial cells, AGE–RAGE signaling increases Nox1 activity; promotes mitochondrial dysfunction; and activates protein kinase C, NF-κB, and mitogen-activated protein kinase (MAPK) pathways, coupling ROS generation to pro-inflammatory and profibrotic responses [126,127]. In companion-animal nutrition, commercial dry and wet pet foods subjected to high-temperature extrusion or retort sterilization contain markedly elevated AGE concentrations relative to unprocessed ingredients [49,56], and RAGE expression has been confirmed in canine and feline vascular and renal tissues, raising the possibility that chronic dietary AGE exposure contributes to oxidative vascular and renal pathology in aging dogs and cats [128,129].

#### 2.2.2. Acrylamide and Its Metabolites as Inducers of Oxidative Stress

Acrylamide is a low-molecular-weight α,β-unsaturated carbonyl compound formed during high-temperature processing of carbohydrate-rich foods, primarily through reactions between asparagine and reducing sugars; it is now a ubiquitous contaminant in fried, baked, and roasted products linked experimentally to neurotoxicity, reproductive toxicity, and carcinogenicity [130,131]. A significant component of this toxicity is oxidative: dietary acrylamide induces dose-dependent increases in ROS, lipid peroxidation, and 8-OHdG in neural, hepatic, and reproductive tissues alongside reduced glutathione and antioxidant enzyme activity [132]. Acrylamide is epoxidized in vivo by cytochrome P450 2E1 (CYP2E1) to glycidamide, a metabolite considerably more potent in forming hemoglobin and DNA adducts; both compounds induce mitochondrial membrane depolarization and caspase-dependent apoptosis in Leydig and Sertoli cells, indicating that mitochondrial ROS generation underlies acrylamide-associated reproductive toxicity [132]. Pharmacological antioxidant supplementation with N-acetylcysteine attenuates acrylamide-induced ROS accumulation and cell death, indicating that oxidative stress lies upstream of much of the downstream toxicity [131]. In companion-animal nutrition, acrylamide has been detected in commercially available extruded dry kibble at per-kilogram concentrations potentially comparable to or exceeding human dietary exposure [133], yet systematic toxicokinetic and oxidative stress studies in dogs and cats remain limited.

#### 2.2.3. Lipid Oxidation Products, 3-MCPD and Glycidyl Esters

The processing of lipid-rich foods during deep-frying, baking, and oil refining promotes extensive lipid peroxidation, causing thermally processed foods and feeds to accumulate primary lipid hydroperoxides and reactive secondary carbonyls—most notably, 4-HNE and MDA. Unlike peroxidation propagated endogenously within tissues, these compounds are pre-formed in the food matrix and delivered directly to the consumer as an exogenous pro-oxidant load; their downstream adduction of mitochondrial and antioxidant targets, which sustains a self-amplifying cycle of oxidative injury, is detailed in Section 3.4. In companion animals, lipid-rich extruded and canned diets with inadequate antioxidant supplementation are especially susceptible to pre-absorptive oxidation, and elevated plasma MDA and urinary 8-OHdG have been reported in dogs fed oxidized fat-containing diets [121,134].

Within the same processing context, 3-chloro-1,2-propanediol (3-MCPD) and its fatty acid esters, as well as glycidyl esters, are generated during high-temperature treatment and deodorization of edible oils, occurring in refined vegetable oils, frying oils, infant formulas, and processed bakery products. Their downstream toxic mechanisms are distinct: 3-MCPD triggers organ-specific oxidative stress in the kidney, testis, and brain in rodent models, including irreversible overoxidation of redox-sensitive protein DJ-1 at Cys106 at doses only modestly exceeding estimated human dietary exposure, compromising antioxidant and neuroprotective functions associated with Parkinson’s disease and diabetic nephropathy pathogenesis [121]. Glycidyl esters, by contrast, are hydrolyzed in the gastrointestinal tract to release glycidol, a direct-acting genotoxin classified as a probable human carcinogen that alkylates DNA independently of the lipid peroxidation cascade. In pet food manufacturing, refined animal fats and vegetable oils subjected to high-temperature rendering and deodorization have been shown to contain both contaminant classes, and given that fat is a major macronutrient in commercial pet diets, companion animals may represent a population with relatively high per-kilogram exposure.

#### 2.2.4. HCAs, PAHs, and Oxidative Stress

HCAs and PAHs are two structurally distinct but mechanistically related classes of genotoxic PICs generated primarily during high-temperature cooking of proteinaceous foods. HCAs—including 2-amino-1-methyl-6-phenylimidazo [4,5-b]pyridine (PhIP), 2-amino-3,8-dimethylimidazo [4,5-f]quinoxaline (MeIQx), and 2-amino-3-methylimidazo [4,5-f]quinoline (IQ)—form through condensation of creatine/creatinine, amino acids, and reducing sugars at temperatures exceeding 150 °C during grilling, frying, and roasting, while PAHs such as benzo[a]pyrene (BaP) and fluoranthene arise from incomplete combustion and pyrolysis, depositing onto food surfaces from smoke, charring, or direct flame contact [120,122,135]. Both classes require metabolic bioactivation to exert genotoxic and oxidative effects: HCAs undergo CYP1A2-mediated N-hydroxylation to reactive intermediates further esterified to electrophilic nitrenium ions that form DNA adducts, a process inherently coupled to ROS generation as a by-product of CYP-mediated metabolism, compounded by HCA-induced glutathione depletion [131]. PAHs undergo a parallel bioactivation cascade—CYP1A1/1B1 epoxidation to DNA-adduct-forming diol epoxides, alongside an aldo-keto reductase pathway generating orthoquinones that undergo sustained one-electron redox cycling independent of adduct formation—with BaP exposure additionally inducing Nrf2-driven antioxidant responses and mitochondrial dysfunction [119,136].

The dietary exposure relevance of HCAs and PAHs extends directly to companion animals: commercially processed extruded and retort-sterilized pet foods have been shown to contain measurable concentrations of PhIP, MeIQx, and selected PAHs, with levels in some products approaching those in grilled human food on a per-kilogram basis [121]. Interspecies differences are clinically relevant—dogs possess hepatic CYP1A2 capable of HCA bioactivation, while cats have markedly reduced glucuronidation capacity that may prolong tissue exposure to hydroxylated HCA metabolites—and in Scottish Terriers, epidemiological evidence supports an inverse association between dietary antioxidant (green and yellow–orange vegetable) intake and risk of transitional cell carcinoma of the urinary bladder, consistent with diet-derived oxidative burden in urothelial carcinogenesis [137]. A direct causal link between dietary HCA or PAH exposure and specific companion-animal tumor types has not been established, and controlled intervention studies are needed.

#### 2.2.5. N-Nitroso Compounds and Heme Iron: Processing-Induced Pro-Oxidants from Cured Meat Products

Cured and processed meat products—including ham, sausages, bacon, hot dogs, salami, and chorizo—represent a qualitatively distinct category of dietary pro-oxidant exposure within the processing-induced contaminant (PIC) framework. Unlike the Maillard-derived AGEs and lipid peroxidation products of generic high-temperature processing, the hazards specific to cured meats arise from two mechanistically independent but synergistic pathways: (i) formation of N-nitroso compounds (NOCs), predominantly nitrosamines, from nitrite-based curing additives reacting with meat-derived amines and (ii) heme iron-catalyzed lipid peroxidation in the gastrointestinal tract following ingestion of myoglobin- and hemoglobin-rich meat. Under the acidic conditions of manufacturing and gastric digestion, residual nitrite is protonated to nitrous acid, which reacts with secondary amines from meat protein degradation to yield N-nitrosamines (NAs)—potent carcinogens, including N-nitrosodimethylamine (NDMA), N-nitrosodiethylamine (NDEA), N-nitrosopiperidine (NPIP), and N-nitrosopyrrolidine (NPYR), several of which are classified by International Agency for Research on Cancer (IARC) as probable or possible human carcinogens [138,139]. High-temperature cooking and reheating of nitrite-cured products substantially amplifies NA formation relative to uncooked products, as elevated temperatures accelerate nitrosation kinetics [140].

Both pathways generate ROS as central mechanistic intermediates and operate at physiologically relevant dietary exposure levels. The pro-oxidant mechanism of N-nitrosamines operates primarily through CYP2E1/CYP2A6-mediated α-hydroxylation, generating reactive diazonium ion intermediates whose formation is inherently coupled to ROS generation and compounded by NA-induced glutathione depletion [138]. These electrophiles also alkylate DNA to form the promutagenic O^6^-methylguanine lesion, linking CYP-derived ROS to genotoxic damage. Endogenous NOC formation adds a further route: gastric nitrite, bacterial nitrosating agents, and inducible nitric oxide synthase (iNOS)-derived nitric oxide (NO) in colonocytes sustain an intraluminal NOC burden independent of exogenous NA content, explaining why cured-meat intake correlates with fecal NOC concentrations in humans [141,142].

Thermal processing denatures the globin protein surrounding the heme moiety, and subsequent gastrointestinal digestion releases catalytically active free Fe^2+^, which drives the Fenton reaction (Fe^2+^ + H_2_O_2_ → Fe^3+^ + •OH + OH^−^) to generate A hydroxyl radical in the intestinal lumen, favored by the low pH and H_2_O_2_ of gastric digestion [69,70]. Heme iron further catalyzes lipid peroxidation—abstracting hydrogen from PUFAs to initiate the radical chain and decomposing lipid hydroperoxides to propagate it—producing MDA and 4-HNE well above pre-consumption levels; these aldehydes are absorbed into the portal circulation, imposing a systemic pro-oxidant burden [69,71].

The IARC classified processed meat as a Group 1 human carcinogen in 2015, and cured meat consumption is now recognized as a meaningful contributor to colorectal, gastric, and hepatocellular cancer risk, in which oxidative DNA damage and ROS-mediated mucosal injury play causal roles [143]. The two pathways are mechanistically intertwined: heme iron promotes bacterial reduction of nitrate to nitrite in the oral cavity and colon, amplifying endogenous NOC formation, so the pro-oxidant and genotoxic hazards cannot be attributed to the curing agent or heme content in isolation [142].

#### 2.2.6. Preservative-Associated Oxidative Toxicants in Processed Foods

Sulfites, benzoates, sorbates, and synthetic antioxidants are widely added to processed foods as preservatives, antioxidants, and anti-browning agents, yet growing evidence indicates that chronic or high-dose exposure to these same compounds can, itself, induce oxidative stress-increased lipid peroxidation, antioxidant enzyme inhibition, glutathione depletion, mitochondrial dysfunction, inflammation, and apoptosis [144,145,146]. These preservatives are ubiquitous in bottled beverages, sauces, baked goods, sausages, canned vegetables, margarines, and commercial pet foods [147,148].

Sodium and potassium sulfites generate ROS and deplete reduced glutathione; in isolated rat hepatocytes, sulfite cytotoxicity was shown to be mediated by free radicals and was prevented by cytochrome P450 inhibition or antioxidant co-treatment [149,150]. Sodium benzoate similarly increases lipid peroxidation and nitric oxide while suppressing antioxidant enzyme activity, with sub-chronic exposure in rats inducing p53 and caspase-3 upregulation consistent with a pro-apoptotic, pro-inflammatory mechanism; under beverage-relevant conditions, benzoate can also react with ascorbic acid to form carcinogenic benzene [145,151]. Potassium sorbate exerts genotoxic effects—chromosomal aberrations, sister-chromatid exchanges, and DNA strand breaks—in human lymphocytes and increases intracellular ROS and p53-associated apoptosis in HepG2 cells [152,153]. Synthetic antioxidants such as butylated hydroxyanisole (BHA), butylated hydroxytoluene (BHT), and tertiary butylhydroquinone (TBHQ) are added to lipid-rich foods to prevent oxidation, yet ethoxyquin—widely used in canned pet food and aquaculture/poultry feed for its low cost and high stability—has, itself, raised oxidative safety concerns [148,154].

Collectively, these findings suggest that food preservation may contribute to oxidative injury through the very additives intended to prevent spoilage, a concern of particular relevance to commercial pet foods, which frequently combine lipid-rich ingredients with long shelf-life requirements [147].

#### 2.2.7. Relevance to Companion Animals

The relevance of these cured-meat-specific pro-oxidant pathways to companion-animal nutrition deserves specific consideration. Although dogs and cats rarely eat cured human foods like ham or sausage as staples, commercial pet products such as jerky-type treats, meat sticks, and retort-processed items using cured pork or beef by-products can expose them to nitrite curing agents and the resulting N-nitrosamines (NAs) at per-kilogram bodyweight levels comparable to human exposures. Furthermore, the heme iron pathway is directly relevant: pet foods and treats made with high-myoglobin materials such as red meat by-products, organ meats, and blood meal supply heme iron that, after thermal processing and digestion, catalyzes postprandial lipid peroxidation in the intestinal lumen of dogs and cats by the same mechanism described in humans [49]. The currently limited characterization of NA concentrations in commercial pet food and treats and the absence of species-specific NA toxicokinetic studies in dogs and cats represent a significant gap in comparative dietary risk assessment that warrants future investigation.

**Table 2:** Processing-induced pro-oxidant compounds in human foods and companion-animal feeds: sources, ROS mechanisms, biomarkers, and species relevance (Section 2.2).

Section	Compound/Factor	Chemical Class	Dietary Source (Human)	Dietary Source (Pet Feed)	Primary ROS-Generating Mechanism	Key Oxidative Biomarkers	Species Relevance & Key References
**2.2. Processing-Induced Contaminants (PICs)**							
2.2	Advanced glycation end-products (AGEs); CML, CEL, MG-H1 (**5**)	Maillard reaction products (protein/lipid + reducing sugar at high temperature)	Roasted and fried meats, baked goods, and coffee; ~10% bioavailability; absorbed fractions accumulate in tissues	Extruded dry kibble and retort-sterilized wet food (markedly elevated vs. unprocessed ingredients; RAGE expressed in canine and feline vascular/renal tissues)	AGE-RAGE interaction → Nox1/Nox2 activation → O_2_•^−^; receptor-independent: AGE-metal chelation → Fenton •OH; PKC-β phosphorylates p47phox → sustained NADPH oxidase; NF-κB → Nox1 transcription upregulation; mitochondrial dysfunction	Plasma CML ↑; tissue AGE fluorescence ↑; vascular O_2_•^−^ (DHE); 8-OHdG ↑; serum sRAGE; Nox2 activity	[56,124,125,126,127,155]
2.2	Acrylamide & glycidamide (CYP2E1 metabolite) (**6**)	α,β-unsaturated amide; epoxide metabolite	Fried potatoes, coffee, biscuits, and bread crust (asparagine + reducing sugars ≥ ~120 °C, Maillard type)	Extruded dry kibble (measurable concentrations; per kg BW exposure may rival human dietary intake)	CYP2E1 epoxidation → glycidamide (more reactive); mitochondrial membrane depolarization → O_2_•^−^; direct GSH conjugation → GSH depletion; NF-κB + Nrf2 co-activation; CYP2E1 metabolic uncoupling → superoxide	Plasma MDA ↑; 8-OHdG ↑; GSH ↓; SOD/CAT/GPx activity ↓; Hb adducts (AA-Val, GA-Val) as exposure biomarkers	[37,130,131,132,156,157]
2.2	Secondary lipid oxidation aldehydes 4-HNE, MDA (processing-amplified) (**7**)	α,β-unsaturated aldehydes (secondary LOPs generated/amplified by thermal processing)	Deep-fried foods, baked goods, and thermally oxidized cooking oils; high-PUFA processed meats	Extruded and canned pet diets with rendered animal fat; oxidized kibble after bag opening (inadequate antioxidant supplementation)	4-HNE: Michael adducts on Complex I NDUFV1 → ETC uncoupling → ↑ mitochondrial O_2_•^−^; adducts inactivate thioredoxin reductase → impaired Prx system; MDA: M_1_dG DNA adducts (mutagenic); both activate NF-κB via redox signaling	Plasma MDA/TBARS ↑; 4-HNE-protein adducts; 8-isoprostane ↑; urinary 8-OHdG ↑; M_1_dG adducts (liver, colon in rodent models)	[25,87,93,94,148,158,159,160]
2.2	3-MCPD & fatty acid esters (**8**)	Chloropropanol process contaminant and its fatty acid esters	Refined vegetable/frying oils, infant formula, smoked meats, and processed bakery products; deodorization of edible oils	Refined animal and vegetable fats in pet food (rendering + high-temperature deodorization); high dietary fat content → elevated per kg exposure	Organ-specific OS (kidney, testis, brain): Hmox1 upregulation; irreversible DJ-1 Cys106 sulfonylation → impaired Nrf2/ARE inducibility (↓ *Hmox1*, *NQO1*, *GCLC*, *GPx2*); GSH depletion; Keap1 release → further Nrf2 nuclear translocation failure	*Hmox1* mRNA ↑ (renal, neural tissue); DJ-1 Cys106 overoxidation; GSH ↓; ROS (DHE/DCFH-DA); PD-associated neuropathological markers	[134,161,162,163,164,165]
2.2	Glycidyl esters → glycidol (**9**)	Epoxide (probable human carcinogen; International Agency for Research on Cancer (IARC) Group 2A)	Refined palm oil, baked goods with palm fat, margarine, and infant formula	Deodorized plant and animal oils in pet food formulation	GI hydrolysis releases glycidol → alkylates DNA → oxidative DNA strand breaks & 8-OHdG; mechanistically distinct from lipid peroxidation cascade; cytochrome P450-independent genotoxicity	Glycidol-Hb adducts (GLYC-Val); 8-OHdG ↑; γ-H2AX (DNA double-strand break marker); micronucleus induction	[163,166,167,168,169,170]
2.2	Heterocyclic aromatic amines (HCAs) PhIP, MeIQx, IQ, harman, and norharman (**10**)	Heterocyclic amines (condensation of creatine/creatinine + amino acids + reducing sugars, ≥ ~150 °C)	Pan-fried, grilled, and roasted meat and fish; formed from Cre + AAs at high temperatures; harman/norharman from carbohydrates	Meat-containing extruded kibble and retort pet food (PhIP, MeIQx detected; per kg levels approaching grilled human food)	CYP1A2 N-hydroxylation → N-OH-HCA → SULT/NAT esterification → nitrenium ions + O_2_•^−^/H_2_O_2_ (metabolic uncoupling); GSH depletion via conjugation; NF-κB activation; SOD/CAT direct inhibition; mitochondrial membrane potential collapse → cytochrome c release	8-OHdG ↑ (colon, liver, reproductive tissues); MDA ↑; SOD/CAT/GPx ↓; urinary HCA metabolites (humans & dogs); HCA-DNA adducts (GC/MS-MS)	[171,172,173,174,175,176]
2.2	Polycyclic aromatic hydrocarbons (PAHs) BaP, fluoranthene, and chrysene (**11**)	PAHs (incomplete combustion and pyrolysis of lipids and proteins; deposition from smoke/flame)	Charred, smoked, and grilled meats and fish; wood/charcoal smoke-exposed foods; direct flame contact	Meat-based extruded kibble and smoked pet treats (selected PAHs detected)	CYP1A1/1B1 epoxidation → arene oxides → diol epoxides → DNA adducts; AKR1A1/1C-mediated orthoquinone → cyclic one-electron redox cycling → sustained O_2_•^−^; AhR-aryl hydrocarbon receptor nuclear translocator (ARNT) → CYP1A1 ↑ + Nox upregulation + Nrf2 target gene suppression (competitive coactivator sequestration); mitochondrial membrane depolarization → cytochrome c release	BaP-DNA adducts; 8-OHdG ↑; MDA ↑; protein carbonyls ↑; *Hmox1*/*NQO1* induction (ARE); F_2_-isoprostane ↑; urinary 1-hydroxypyrene (PAH exposure biomarker)	[177,178,179,180,181,182,183]
2.2	Preservatives (sulfites, benzoates, sorbates, and synthetic antioxidants) (**12**)	Food preservatives, antioxidant stabilizers, and anti-browning agents	Bottled soft drinks, fruit juices, sauces, gravies, bread/pizza dough, sausages, canned vegetables, margarines, and processed lipid-rich foods	Commercial pet foods, canned pet food, and aquaculture and poultry feed formulations	Sulfites → ROS formation + lipid peroxidation + GSH/GSSG depletion; benzoates → ROS overproduction + SOD/CAT inhibition + inflammatory signaling (IL-6/TNF-α) + apoptosis; sorbates → intracellular ROS + oxidative DNA strand breaks + p53-mediated apoptosis; synthetic antioxidants (BHA/BHT/TBHQ/ethoxyquin) → redox imbalance + mitochondrial oxidative stress	TBARS/MDA ↑; ROS ↑; GSH ↓; SOD/CAT/GPx ↓; NO ↑; IL-6/TNF-α ↑; DNA strand breaks; micronucleus formation; caspase-3 ↑; p53 ↑	[148,149,151,152,153,184,185,186,187,188,189,190]

Abbreviations: PICs, processing-induced contaminants; ROS, reactive oxygen species; AGEs, advanced glycation end-products; CML, Nε-carboxymethyllysine; CEL, Nε-carboxyethyllysine; MG-H1, methylglyoxal-derived hydroimidazolone 1; RAGE, receptor for advanced glycation end-products; Nox, NADPH oxidase; O_2_•^−^, superoxide anion radical; •OH, hydroxyl radical; NF-κB, nuclear factor kappa B; CYP2E1, cytochrome P450 family 2 subfamily E member 1; GSH, reduced glutathione; Nrf2, nuclear factor erythroid 2-related factor 2; SOD, superoxide dismutase; CAT, catalase; GPx, glutathione peroxidase; 8-OHdG, 8-hydroxy-2′-deoxyguanosine; 4-HNE, 4-hydroxy-2-nonenal; MDA, malondialdehyde; TBARS, thiobarbituric acid-reactive substances; 3-MCPD, 3-monochloropropane-1,2-diol; Hmox1, heme oxygenase 1; ARE, antioxidant response element; IARC, International Agency for Research on Cancer; HCAs, heterocyclic aromatic amines; PhIP, 2-amino-1-methyl-6-phenylimidazo [4,5-b]pyridine; MeIQx, 2-amino-3,8-dimethylimidazo [4,5-f]quinoxaline; IQ, 2-amino-3-methylimidazo [4,5-f]quinoline; PAHs, polycyclic aromatic hydrocarbons; BaP, benzo[a]pyrene; AhR, aryl hydrocarbon receptor; ARNT, aryl hydrocarbon receptor nuclear translocator; DHE, dihydroethidium; DCFH-DA, 2′,7′-dichlorodihydrofluorescein diacetate; NO, nitric oxide; IL-6, interleukin-6; TNF-α, tumor necrosis factor α; BHA, butylated hydroxyanisole; BHT, butylated hydroxytoluene; TBHQ, tert-butylhydroquinone. → indicates mechanistic relationships; ↑ indicates an increase; ↓ indicates a decrease.

## 3. Mechanisms of ROS Generation by Food- and Feed-Derived Toxicants

The preceding section described the formation, dietary sources, and species-specific exposure profiles of major PICs. The present section focuses on the molecular and cellular mechanisms through which these structurally diverse compounds generate ROS and disrupt cellular redox homeostasis. Despite their chemical heterogeneity, PICs converge on a limited set of ROS-generating pathways, which are organized here by mechanism rather than by compound class: receptor-mediated NADPH oxidase activation, cytochrome P450-dependent redox cycling, mitochondrial electron transport impairment, the lipid peroxidation cascade, and progressive depletion of endogenous antioxidant defenses. These pathways are not mutually exclusive; rather, they interact in a feed-forward manner to sustain oxidative stress well beyond the initial exposure event.

### 3.1. Receptor-Mediated ROS Signaling

Membrane receptor–NADPH oxidase axes constitute a primary mechanism by which extracellular ligands transduce signals into intracellular ROS production. The AGE–RAGE–Nox axis represents the best-characterized example: ligand engagement of RAGE activates Nox1- and Nox2-containing NADPH oxidase complexes at the plasma membrane, leading to vectorial superoxide release into the extracellular space and, following dismutation, to intracellular hydrogen peroxide accumulation [124,126,127]. At the molecular level, RAGE signaling proceeds through Rac1-dependent assembly of Nox2 cytosolic subunits p47phox and p67phox at the membrane-bound gp91phox-p22phox heterodimer; PKC-β isoforms phosphorylate p47phox, lowering the activation threshold and sustaining NADPH oxidase activity even after ligand withdrawal. In vascular smooth muscle and mesangial cells, this pathway is amplified by concurrent RAGE-mediated upregulation of Nox1 transcription via NF-κB, creating a positive feedback loop in which ROS-activated NF-κB further induces Nox1 expression [136].

A parallel receptor-mediated pathway is initiated by the aryl hydrocarbon receptor (AhR) upon binding of PAH ligands such as benzo[a]pyrene. AhR-ARNT nuclear translocation drives transcriptional upregulation of CYP1A1 and, critically, of NADPH oxidase subunits, establishing a link between xenobiotic sensing and oxidase-dependent superoxide generation that is mechanistically distinct from the enzymatic ROS produced during CYP-mediated bioactivation (discussed in Section 3.2). AhR activation additionally suppresses the expression of Nrf2 target genes through competitive sequestration of shared coactivators, thereby simultaneously enhancing ROS production and impairing compensatory antioxidant responses [182].

### 3.2. Cytochrome P450-Dependent ROS Production

Cytochrome P450 enzymes, particularly the CYP1 family, are central mediators of PIC bioactivation and constitute a major enzymatic source of ROS. CYP-catalyzed monooxygenation proceeds through a cycle in which molecular oxygen is activated by a ferrous-oxy complex; uncoupling of this cycle—whereby electrons are transferred to O_2_ without substrate hydroxylation—releases superoxide directly at the active site. The extent of uncoupling depends on the fit between the substrate and the enzyme active site, and several PIC metabolites are poor substrates that promote uncoupling rather than productive oxidation [191].

HCAs undergo CYP1A2-mediated N-hydroxylation to yield reactive N-hydroxy intermediates, and PAHs are epoxidized by CYP1A1 and CYP1B1 to arene oxides and subsequently to diol epoxides [192]. A critical downstream ROS-generating mechanism is quinone redox cycling: PAH diol epoxides are reduced by aldo-keto reductases (AKR1A1 and AKR1C) to catechols that spontaneously oxidize to orthoquinones; these quinones accept single electrons from NADPH or NADH via a semiquinone radical intermediate, then re-donate the electron to O_2_, regenerating the quinone and producing superoxide in a repetitive, substrate-independent cycle [183]. This redox cycling is self-sustaining as long as reductant supply persists and can generate ROS burdens that greatly exceed those attributable to the initial bioactivation step alone. In neuronal cell models exposed to HCAs including IQ-type compounds, concentration-dependent ROS elevation, glutathione depletion, and superoxide dismutase inhibition co-occur with cytochrome c release, indicating that CYP-derived and mitochondrial ROS are generated in parallel [192,193].

### 3.3. Mitochondrial Dysfunction

Mitochondrial electron transport chain (ETC) impairment is a convergent downstream consequence of exposure to multiple PIC classes and represents a self-amplifying source of ROS. Under physiological conditions, approximately 0.1–2% of electrons leaking from Complexes I and III are captured by O_2_ to form superoxide at the flavin mononucleotide site of Complex I and the ubiquinone binding site of Complex III. Adduction or inhibition of Complex I subunits by electrophilic PICs—including 4-HNE Michael adducts on the NADH: ubiquinone oxidoreductase core subunit V1 (NDUFV1) subunit and acrylamide-derived adducts on lipoic acid-containing subunits of the pyruvate dehydrogenase complex—increases electron leakage at these sites, amplifying superoxide output in direct proportion to the degree of ETC disruption [194].

Membrane depolarization further uncouples oxidative phosphorylation from ATP synthesis and, paradoxically, increases the proportion of partially reduced ubisemiquinone available for single-electron O_2_ reduction. Acrylamide and its metabolite, glycidamide, induce the collapse of mitochondrial membrane potential in reproductive cells [132]; PAH-derived electrophiles similarly promote cytochrome c release in cardiac and pulmonary models, establishing a positive feedback loop in which ROS-mediated lipid peroxidation of the inner mitochondrial membrane cardiolipin further destabilizes the ETC and amplifies electron leakage [195]. In companion animals, elevated plasma malondialdehyde and urinary 8-OHdG in dogs fed oxidized fat-containing diets are consistent with a contribution of diet-derived ETC inhibitors to sustained mitochondrial ROS production in vivo.

### 3.4. Lipid Peroxidation Cascade

When antioxidant defenses are depleted by other PIC mechanisms, endogenous peroxidation of tissue PUFAs proceeds unchecked, adding to the pre-formed aldehyde load delivered by processed diets [121] (Section 2.2.3). The toxicity of these aldehydes lies in the electrophilicity of 4-HNE, a bifunctional electrophile that forms Michael adducts with Cys, His, and Lys residues; beyond the Complex I NDUFV1 adduction that couples lipid peroxidation to ETC-derived ROS (Section 2.2.3 and Section 3.3), 4-HNE additionally adducts and inactivates the thioredoxin reductase active-site selenocysteine, impairing the thioredoxin system and thereby reducing the capacity for peroxiredoxin (Prx)-mediated peroxide clearance [134]. MDA is both a biomarker and an active contributor to cross-linking of proteins and nucleic acids, generating exocyclic DNA adducts (M_1_dG) that are mutagenic and have been detected in hepatic and colonic tissues of rodents chronically exposed to dietary lipid oxidation products.

### 3.5. Antioxidant Depletion

Progressive depletion of endogenous antioxidant defenses amplifies ROS accumulation from all upstream sources and constitutes the final converging mechanism by which factors in unprocessed foods and PICs sustain oxidative stress. GSH depletion occurs through multiple simultaneous routes: direct conjugation of electrophilic PICs (acrylamide, 4-HNE, and HCA N-hydroxy metabolites) with GSH via glutathione S-transferases consumes the GSH pool; oxidation of GSH to oxidized glutathione (GSSG) by GPx during peroxide detoxification requires NADPH-dependent reduction by glutathione reductase, and NADPH depletion by concurrent CYP activity limits regeneration capacity. When the GSH/GSSG ratio falls below approximately 10:1, the redox buffer is effectively exhausted, and thiol-dependent enzymes including protein tyrosine phosphatases and GAPDH are susceptible to oxidative inactivation [119]. Superoxide dismutase (SOD) and catalase activities are reduced by dietary acrylamide, HCAs, and PAHs in multiple rodent models, reflecting both transcriptional suppression via NF-κB-mediated repression of Mn-SOD and direct oxidative inactivation of the enzyme active sites. Redox-sensing protein DJ-1 (PARK7), whose antioxidant function depends on the nucleophilic Cys106 residue, is irreversibly over-oxidized to the sulfonyl form upon 3-MCPD exposure, abrogating its neuroprotective and chaperone activities at doses approaching estimated dietary intake levels [121]. This last point is mechanistically significant because DJ-1 normally acts as a sensor that promotes Nrf2 nuclear translocation by sequestering Kelch-like ECH-associated protein 1 (Keap1); its inactivation therefore impairs the inducibility of the entire Nrf2/ARE-driven antioxidant gene battery—including Hmox1, NAD(P)H quinone oxidoreductase 1 (NQO1), GCLC, and GPx2—at the very moment that the oxidant burden is highest.

Considered together, these five mechanisms—receptor-mediated NADPH oxidase activation, CYP-dependent redox cycling, mitochondrial ETC impairment, the lipid peroxidation cascade, and antioxidant depletion—operate in a mutually reinforcing manner. Each individual PIC typically engages two or more of these pathways simultaneously, and the co-occurrence of multiple PICs in thermally processed diets creates a combinatorial oxidant burden whose magnitude likely exceeds simple additivity. Redox-sensitive transcription factors NF-κB, Nrf2/ARE, and activator protein 1 (AP-1) integrate signals from all five pathways and translate sustained oxidative stress into inflammatory gene expression and adaptive or maladaptive antioxidant responses; because these transcriptional programs are broadly conserved across mammalian species, the mechanistic framework developed in this section is applicable to both human dietary exposure and to companion animals chronically consuming thermally processed pet foods.

### 3.6. Antioxidant Gene Induction as a Biomarker of Pro-Oxidant Stress Imposed by Dietary Compounds

The five ROS-generating mechanisms described in Section 3.1, Section 3.2, Section 3.3, Section 3.4 and Section 3.5 are not restricted to exogenous PIC-class toxicants; they apply equally to a broad range of redox-active molecules generated endogenously or introduced via diet. This principle is most clearly illustrated by the Keap1–Nrf2–ARE axis. Under basal conditions, Nrf2 is sequestered in the cytoplasm by Keap1, which presents it to a Cullin 3-based E3 ubiquitin ligase complex for proteasomal degradation. When electrophilic compounds modify critical sensor cysteine residues on Keap1 (C151, C273, and C288), ubiquitination is disrupted, and Nrf2 translocates to the nucleus, where it drives expression of a cytoprotective gene battery including GCL, GSTs, heme oxygenase-1 (HO-1), *NQO1*, thioredoxin reductase (TrxR), and ferritin [196,197].

The critical interpretive point is that the modification of Keap1 cysteine residues is, itself, direct molecular evidence that the compound in question is imposing electrophilic and oxidative stress on the cell. This reaction is chemically indistinguishable from the cysteine adduction caused by 4-HNE described in Section 3.4 and follows the same chemical logic as protein modification by lipid peroxidation by-products. Accordingly, Keap1 modification by dietary electrophiles (polyphenols and isothiocyanates) and by endogenous lipid peroxidation products—including 4-HNE and its structural analog, 4-hydroxy-2-hexenal (4-HHE), generated from omega-3 PUFA oxidation—belong to the same mechanistic category.

From this perspective, induction of the Nrf2/ARE gene battery (including *Hmox1*, *NQO1*, *GCLC*, and *GPx2*) as described in Section 3.5 should be interpreted not as a straightforward protective adaptation but as a hormetic compensatory response to preceding or concurrent oxidative and electrophilic injury [198]. The hormesis model—in which a low-level toxic challenge transiently elevates cellular defense capacity above baseline—applies equally to the induction of mitochondrial antioxidant enzymes, including manganese superoxide dismutase (MnSOD) and thioredoxin 2 (TRX2), via the sirtuin 1 (SIRT1)/peroxisome proliferator-activated receptor gamma coactivator 1 α (PGC-1α) axis following ETC perturbation as described in Section 3.3: compounds that disrupt the mitochondrial electron transport chain first transiently increase superoxide generation at Complex I/III, and the resulting ROS signal activates AMP-activated protein kinase (AMPK)/SIRT1 to initiate the biogenesis and antioxidant response.

This integrated mechanistic framework carries direct implications for species-specific risk assessment. The Nrf2/ARE axis is broadly conserved across mammalian species; however, interspecies differences in phase II metabolic capacity and GSH biosynthetic reserve determine the threshold between adaptive hormesis and cytotoxic pro-oxidant injury. Cats are particularly vulnerable, owing to their limited glucuronidation capacity and reduced GCL activity [199,200], and are therefore at greater risk of transitioning from the hormetic domain into the cytotoxic domain following exposure to redox-active dietary compounds that elicit only adaptive responses in dogs or humans. The compound-specific evidence supporting this framework is detailed in Section 6.

## 4. ROS-Associated Diseases Linked to Food and Feed Exposure

**Figure 3:**
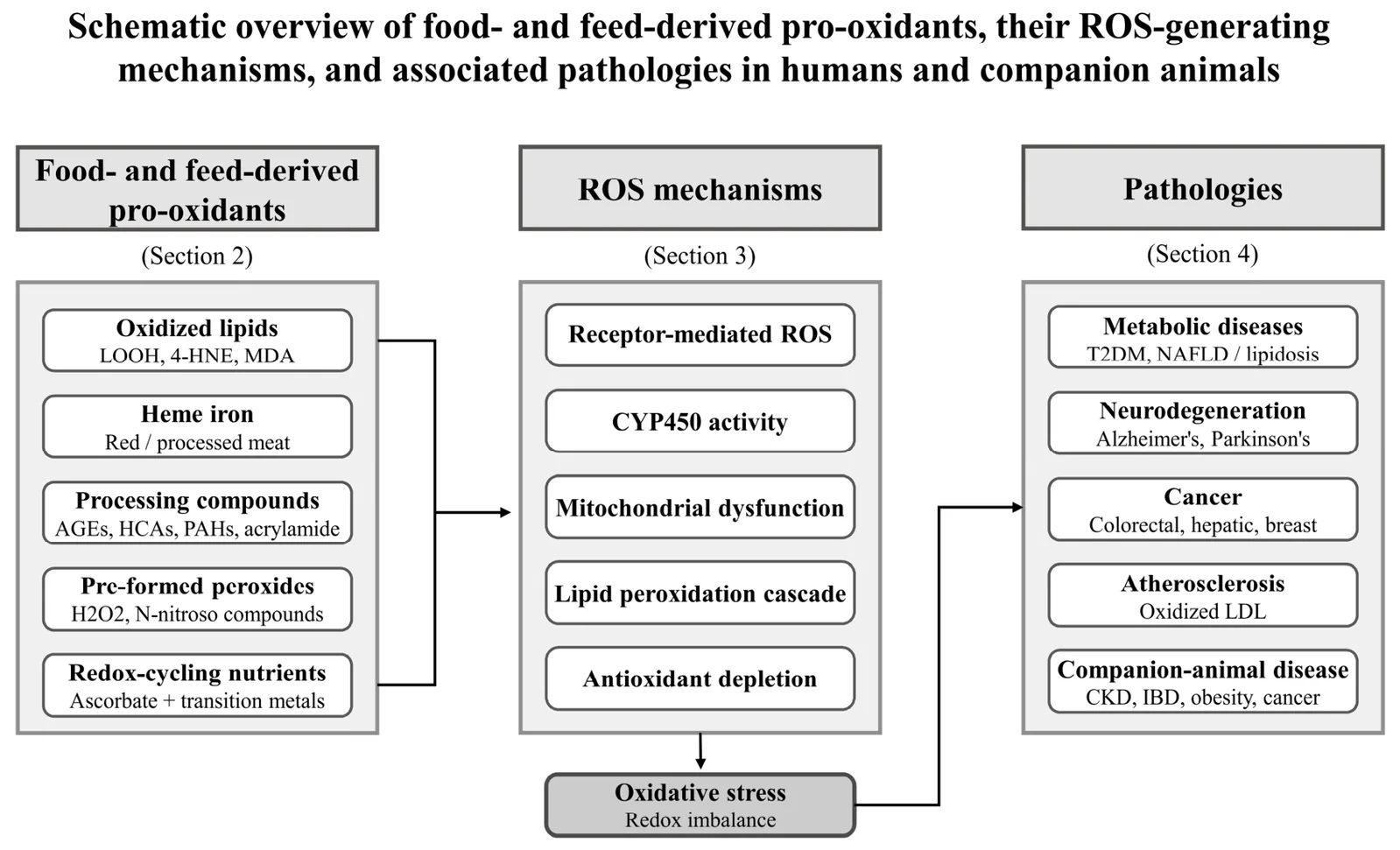
Food- and feed-derived pro-oxidants (Section 2) drive interconnected ROS-generating mechanisms (Section 3) that converge on a shared state of oxidative stress, producing parallel disease processes in humans and companion animals (Section 4). Arrows indicate the direction of the proposed mechanistic relationships.

The preceding sections have established that both human diets and companion-animal feeds contain a spectrum of exogenous ROS and processing-induced pro-oxidant compounds that gain systemic access following ingestion. The convergent mechanisms through which these dietary oxidants impair cellular redox homeostasis—receptor-mediated NADPH oxidase activation, CYP-dependent redox cycling, mitochondrial electron transport chain (ETC) impairment, propagating lipid peroxidation cascades, and progressive antioxidant depletion—are not mere biochemical curiosities; they translate into clinically meaningful, organ-specific pathology across multiple disease categories. The major pathways linking food- and feed-derived pro-oxidants to oxidative stress and associated pathologies are summarized in Figure 3. In this section, we review the evidence linking dietary ROS exposure to the major chronic disease entities that affect both humans and companion animals, organized by organ system and disease category.

### 4.1. Metabolic Diseases

#### 4.1.1. T2DM

T2DM is a disease of impaired redox homeostasis superimposed on a background of nutrient excess. The global prevalence of T2DM has escalated dramatically over recent decades, with approximately 9.3% of the adult population (20–79 years) affected as of 2019, a figure projected to rise substantially in parallel with the continued global adoption of processed, calorie-dense dietary patterns [201]. Chronic hyperglycemia, the defining metabolic lesion of T2DM, perpetuates and amplifies oxidative stress through at least four distinct pathways that is each directly modulated by dietary substrate availability: (i) glucose auto-oxidation, which generates superoxide and hydrogen peroxide non-enzymatically in proportion to glucose concentration; (ii) activation of the polyol pathway, wherein aldose reductase reduces excess glucose to sorbitol at the expense of NADPH, depleting the principal cofactor required for glutathione regeneration; (iii) enhanced formation of AGEs from dietary and endogenously formed reducing sugars, which engage RAGE to activate Nox1/Nox2-dependent superoxide production in endothelial, mesangial, and immune cells; and (iv) activation of protein kinase C (PKC) isoforms by diacylglycerol, which phosphorylates p47phox to lower the NADPH oxidase activation threshold [202]. At the cellular level, these pathways converge on mitochondrial dysfunction: excessive nutrient flux through the ETC generates a surplus of reducing equivalents, increasing electron leakage at Complexes I and III and thereby augmenting superoxide production in direct proportion to the degree of metabolic overload [203]. The resulting ROS activates the stress-sensitive kinase c-jun N-terminal kinase (JNK), which phosphorylates insulin receptor substrate-1 (IRS-1) at serine residues, attenuating insulin signaling and establishing a self-reinforcing cycle between oxidative stress and insulin resistance [204]. In pancreatic β-cells, which express comparatively low levels of catalase and glutathione peroxidase relative to other cell types, this ROS burden is particularly consequential: acute ROS generation potentiates glucose-stimulated insulin secretion (GSIS) through redox-sensitive signaling, but chronic oxidative loading impairs β-cell function and triggers mitochondrial apoptosis, progressively eroding insulin secretory capacity [205].

The dietary dimension of T2DM-associated oxidative stress is particularly salient in the context of ultra-processed food consumption. High-temperature processed foods contribute directly to the dietary AGE burden, which is estimated to account for approximately 10% of absorbed AGEs deposited in tissues [21]. Dietary fructose and refined sugars accelerate the Maillard reaction in vivo, amplifying endogenous AGE formation and RAGE signaling. Conversely, dietary patterns rich in antioxidant phytochemicals—including flavonoids, carotenoids, curcumin, and gallic acid—have been shown to prevent oxidative stress and reduce ROS-driven downstream complications in T2DM through activation of Nrf2/ARE-mediated antioxidant gene expression and modulation of redox-sensitive signaling pathways including Keap1, PKC, and IκB kinase-β [206]. The progressive impairment of endogenous antioxidant enzyme activities—including reduced SOD, CAT, GPx, and glutathione reductase—documented across multiple clinical studies in T2DM patients and rodent models further underscores the self-reinforcing nature of diet-induced oxidative pathology in metabolic disease [207].

#### 4.1.2. Nonalcoholic Fatty Liver Disease and Hepatic Lipidosis

Nonalcoholic fatty liver disease (NAFLD) has emerged as the most prevalent chronic liver condition globally, now estimated to affect approximately 25% of the adult population worldwide, with a substantially higher prevalence among individuals with obesity and T2DM [208]. The pathogenesis of NAFLD encompasses a spectrum from simple hepatic steatosis through nonalcoholic steatohepatitis (NASH), fibrosis, cirrhosis, and hepatocellular carcinoma (HCC), and oxidative stress has been positioned as the central mechanistic driver of disease progression across this continuum, constituting the primary pathogenic insult in the updated ‘multiple-hit’ hypothesis of NAFLD development [209].

Hepatic lipid accumulation, the initiating ‘first hit,’ sensitizes hepatocytes to oxidative injury by impairing the function of multiple ROS-generating organelles simultaneously. Mitochondrial β-oxidation of fatty acids is initially upregulated as a compensatory response to lipid overload, but this adaptation becomes bioenergetically inefficient over time, and the resulting increase in ETC activity markedly amplifies electron leakage at Complexes I and III, generating superoxide that exceeds the mitochondrial antioxidant buffering capacity [210]. The endoplasmic reticulum (ER) constitutes a parallel source of ROS in NAFLD: ER stress activates the unfolded protein response (UPR), which induces ERO1α-mediated oxidative protein folding that produces hydrogen peroxide as a direct by-product while simultaneously disrupting Ca^2+^ homeostasis and further impairing mitochondrial function through mitochondria-associated membrane (MAM) crosstalk [211]. NADPH oxidases, particularly Nox2 expressed on hepatic Kupffer cells and activated by dietary lipopolysaccharide (LPS) derived from gut microbiota dysbiosis, contribute a third major source of hepatic ROS [209].

The dietary linkage to NAFLD oxidative stress is mechanistically explicit. Excessive intake of saturated fatty acids—abundant in processed meat products and tropical fat-containing processed foods—promotes hepatocyte lipotoxicity through the formation of ceramides and diacylglycerols (DAGs), electrophilic lipid species that impair mitochondrial function and activate pro-inflammatory kinase cascades. Dietary fructose, present at high concentrations in sugar-sweetened beverages and confectionery, is preferentially converted to hepatic lipid through de novo lipogenesis; H_2_O_2_ generated during this process directly upregulates SREBP1c-mediated lipogenic gene expression, establishing a positive feedback loop between ROS production and hepatic lipid accumulation [212,213]. Dietary oxidative balance score (OBS) studies in large cross-sectional cohorts have demonstrated that the cumulative pro- vs. anti-oxidant balance of the diet-integrating consumption of fruits, vegetables, whole grains, and antioxidant vitamins against processed food, alcohol, and iron intake-is an independent predictor of NAFLD prevalence and advanced fibrosis risk after adjustment for body mass index and other confounders [212]. Among validated oxidative stress biomarkers in NAFLD, MDA, 4-HNE, 8-isoprostane, 8-OHdG, and nitrotyrosine are consistently elevated in clinical NASH relative to simple steatosis or healthy controls, while antioxidant enzyme activities (SOD, CAT, GPx, and paraoxonase-1) are correspondingly reduced [214].

### 4.2. Neurodegenerative Diseases

The central nervous system (CNS) is uniquely susceptible to oxidative injury by virtue of its high metabolic rate, abundant polyunsaturated fatty acid content in membrane phospholipids, and relatively modest antioxidant enzyme capacity compared with peripheral tissues, as well as the post-mitotic nature of neurons, which precludes regenerative replacement of oxidatively damaged cells [215]. Accordingly, oxidative stress has emerged as a convergent pathogenic mechanism shared across the major neurodegenerative disorders, with dietary pro-oxidant exposure constituting a potentially modifiable upstream contributor to neuronal ROS burden.

#### 4.2.1. Alzheimer’s Disease

Alzheimer’s disease (AD) is the most prevalent neurodegenerative disorder and the leading cause of dementia, pathologically defined by extracellular amyloid-β (Aβ) plaques and intracellular neurofibrillary tangles (NFTs) of hyperphosphorylated tau protein. A substantial body of post-mortem, biomarker, and experimental evidence positions oxidative stress as both an early event and an amplifying factor in AD pathogenesis. Markers of oxidative damage—including elevated 4-HNE and MDA (lipid peroxidation), protein carbonyls and nitrotyrosine (protein oxidation), and 8-OHdG (DNA oxidation)—are detectable in the hippocampus and temporal cortex of AD patients at stages preceding significant plaque or tangle pathology, indicating that redox dysregulation is not merely a downstream consequence of neurodegeneration but an early contributor to the cascade [13,216].

Mechanistically, Aβ oligomers directly engage membrane lipids to generate reactive carbonyl species through metal-catalyzed oxidation, and transition metals such as copper and iron concentrated within amyloid plaques catalyze Fenton-type hydroxyl radical generation that damages adjacent neurons [217]. Hyperphosphorylated tau, in turn, promotes mitochondrial dysfunction by associating with complex I subunits, increasing electron leakage and superoxide generation within tau-affected neurons. The resulting oxidative modification of mitochondrial complex I—including 4-HNE adduction of the NDUFV1 subunit—impairs ATP production and elevates local ROS concentrations, establishing a pathological cycle between tau pathology and mitochondrial oxidative stress [218]. Dietary exposures contribute to this pathway through multiple routes: AGEs derived from thermally processed foods engage neuronal RAGE to activate Nox2-dependent superoxide production and NF-κB-mediated neuroinflammation; dietary trans fats and saturated fatty acids promote mitochondrial dysfunction and insulin resistance in the brain (‘type 3 diabetes’), impairing neuronal glucose metabolism and amplifying ROS production; and dietary deficiencies in antioxidant vitamins C and E, selenium, and polyphenols impair the Nrf2/ARE-mediated neuroprotective antioxidant response [219,220].

Epidemiological evidence supports dietary modulation of AD risk: adherence to Mediterranean and MIND (Mediterranean-DASH Intervention for Neurodegenerative Delay) dietary patterns, characterized by high intake of antioxidant-rich foods and low processed food consumption, has been associated with slower cognitive decline and reduced AD incidence in prospective cohort studies [221]. At the molecular level, dietary polyphenols including resveratrol, curcumin, and epigallocatechin-3-gallate (EGCG) attenuate Aβ-induced ROS generation, activate sirtuin-mediated mitochondrial biogenesis, inhibit RAGE signaling, and upregulate Nrf2-target neuroprotective genes, providing mechanistic support for diet-mediated AD risk modification [220,222].

From a comparative perspective, however, the disease phenotype differs across species, even when oxidative mechanisms overlap. Alzheimer’s disease, with its hallmark Aβ plaques and tau tangles, does not occur naturally in dogs or cats, though aged dogs develop a cognitive dysfunction syndrome (CDS), characterized by Aβ accumulation, neuroinflammation, and oxidative stress-mediated neuronal loss with clinical parallels to early-stage human dementia [223]. This makes the aging dog a naturally occurring large-animal model of early Aβ pathology with significant translational value for the evaluation of the relationship between dietary antioxidant intake and cognitive aging, a relationship that remains difficult to study experimentally in human populations.

#### 4.2.2. Parkinson’s Disease

Parkinson’s disease (PD) is the second most prevalent neurodegenerative disorder, characterized by the selective loss of dopaminergic neurons in the substantia nigra pars compacta (SNpc) and the accumulation of α-synuclein-containing Lewy bodies. The SNpc is intrinsically predisposed to oxidative stress due to its high dopamine metabolic flux: dopamine auto-oxidation generates dopamine quinones and H_2_O_2_, and monoamine oxidase (MAO)-catalyzed dopamine catabolism produces H_2_O_2_ as a direct enzymatic by-product, placing the dopaminergic neuron under constitutive oxidative burden that is further amplified by PD-related mitochondrial Complex I dysfunction and iron accumulation [224].

Dietary exposures contribute to PD-associated oxidative stress through both direct and indirect routes. Chronic dietary exposure to pesticide residues in contaminated produce, particularly organochlorines, organophosphates, and Complex I inhibitor rotenone in naturally occurring food sources, has been epidemiologically and mechanistically linked to PD risk through the induction of mitochondrial ROS production and selective dopaminergic neurotoxicity [225,226]. High dietary meat consumption may also contribute through two mechanisms: elevated saturated fatty acid intake promotes neuroinflammation and mitochondrial dysfunction, and exogenous α-synuclein present in meat products may translocate through the gut–brain axis to seed CNS α-synuclein aggregation, as supported by emerging evidence for a gut-first model of PD pathogenesis [227,228]. Conversely, dietary fiber intake, adherence to the Mediterranean diet, and habitual consumption of fruits and vegetables rich in polyphenols—particularly flavonoids—have been inversely associated with PD risk in prospective studies, consistent with a protective role of dietary antioxidants against the sustained oxidative burden imposed on dopaminergic neurons [229].

Processing-induced contaminants in the diet may additionally contribute to PD-associated neurodegeneration. As discussed in Section 2.2 and Section 3-MCPD—generated during high-temperature refining of edible oils and present in a broad range of processed foods—has been shown to cause irreversible overoxidation of redox-sensing protein DJ-1 at Cys106, impairing its neuroprotective function in a manner mechanistically analogous to the DJ-1 loss-of-function mutations associated with familial early-onset PD [121]. This finding raises the possibility that chronic low-level dietary 3-MCPD exposure may constitute a partial phenocopy of genetic PD susceptibility through ROS-mediated DJ-1 inactivation, providing a plausible dietary—molecular link to sporadic PD pathogenesis.

### 4.3. Cancer

Cancer is among the most extensively documented sequelae of chronic oxidative stress, and the mechanistic links between ROS, dietary pro-oxidant exposure, and carcinogenesis are among the most thoroughly characterized in biomedical literature. Oxidative stress drives malignant transformation through three inter-related mechanisms: (i) direct oxidative DNA damage, generating mutagenic lesions—most prominently, 8-oxo-7,8-dihydro-2′-deoxyguanosine (8-OHdG)—that, if unrepaired prior to replication, produce guanine-to-thymine transversions in proto-oncogenes and tumor suppressor genes; (ii) epigenetic dysregulation, whereby ROS-mediated oxidation of the Ten–Eleven Translocation (TET) enzyme cofactor and CpG dinucleotides alters DNA methylation patterns at cancer-relevant loci; and (iii) redox-driven activation of oncogenic signaling cascades—including NF-κB, STAT3, AP-1, and the phosphoinositide 3-kinase (PI3K)/protein kinase B (Akt)/mechanistic target of rapamycin (mTOR) pathway—that promote proliferation, suppress apoptosis, and facilitate immune evasion [12,230]. The dietary contribution to carcinogenic oxidative stress is mechanistically explicit and epidemiologically compelling across multiple cancer types.

The following subsections examine this dietary oxidative stress–cancer relationship across the major human cancer types—colorectal, hepatocellular, and hormonally driven malignancies. Dietary pro-oxidant-driven carcinogenesis is not unique to humans, however: the parallel evidence implicating HCAs, aflatoxin B1, and oxidative stress in cancer among companion animals and livestock is addressed in Section 4.5.4, and the cross-species mechanistic and epidemiological parallels are synthesized in Section 5.3.

#### 4.3.1. Colorectal Cancer

Colorectal cancer (CRC) is the third most common cancer globally and the one most directly linked to dietary pro-oxidant exposure through both epidemiological and mechanistic evidence. The colonic epithelium is constitutively exposed to luminal pro-oxidants derived from the diet, including heme iron from red and processed meat, secondary bile acids, lipid peroxidation products (4-HNE, MDA), and HCAs and PAHs generated during high-temperature cooking [231]. Heme iron catalyzes lipid peroxidation in the colonic lumen through Fenton-type chemistry, generating cytotoxic and genotoxic aldehydes—particularly 4-HNE—that form promutagenic exocyclic DNA adducts (etheno-adducts) in colonocyte nuclei; this mechanism has been directly confirmed in human ileostomy studies and in rodent models, where dietary heme supplementation reproducibly increases colonocyte 8-OHdG and MDA concentrations and promotes the formation of aberrant crypt foci [232]. HCAs generated during high-temperature processing—most prominently, PhIP and MeIQx—are activated by hepatic CYP1A2 to N-hydroxylated intermediates that are further acetylated by N-acetyltransferase-2 (NAT2) to form reactive nitrenium ions, which form C8-deoxyguanosine adducts in colonic mucosa and have been shown to selectively activate the Wnt/β-catenin oncogenic pathway in rodent and organoid models [233].

In humans, this bioactivation is mediated primarily by hepatic CYP1A2, which converts dietary PhIP and MeIQx into reactive intermediates subsequently O-acetylated by NAT2 to form the mutagenic DNA adducts described above; the rapid-acetylator *NAT2* phenotype confers significantly elevated colorectal cancer risk in the context of high red-meat consumption. The substantial interspecies differences in CYP1A2 activity and phase II conjugation capacity that modulate this bioactivation pathway in dogs and cats are detailed in Section 5.3.

Each 50 g/day increment in processed meat consumption is associated with an approximately 18% increase in CRC risk, an effect magnitude consistent with the genotoxic and pro-oxidant burden imposed by dietary heme, N-nitroso compounds, and HCAs in processed meat products. Conversely, dietary patterns rich in antioxidant phytochemicals—particularly cruciferous vegetables containing sulforaphane, a potent Nrf2 inducer—are inversely associated with CRC risk and have been shown to reduce colonocyte 8-OHdG levels and HCA-DNA adduct formation in controlled intervention studies [234,235].

#### 4.3.2. Hepatocellular Carcinoma

Hepatocellular carcinoma (HCC) represents the malignant endpoint of the NAFLD/NASH disease continuum described in Section 4.1.2, and the role of dietary oxidative stress in its pathogenesis integrates the hepatic ROS mechanisms described in that context with additional genotoxic inputs from dietary contaminants. In the setting of chronic hepatic oxidative stress—established by lipid overload, alcohol, iron excess, or persistent viral hepatitis—ROS-mediated activation of the NF-κB and STAT3 transcription factors in hepatic stellate cells drives fibrogenic cytokine production and the creation of a pro-tumorigenic microenvironment characterized by impaired immune surveillance, chromosomal instability, and dysregulated hepatocyte proliferation [236]. Dietary aflatoxin B1 (AFB1)—a mycotoxin produced by *Aspergillus* species contaminating improperly stored grains, nuts, and legumes in tropical and subtropical regions—is the most potent known dietary hepatocarcinogen: CYP3A4- and CYP1A2-mediated epoxidation of AFB1 generates a highly reactive 8,9-exo-epoxide that forms bulky N7-guanine adducts, producing the characteristic G → T transversion at codon 249 of the *TP53* tumor suppressor gene identified in a high proportion of HCC cases from AFB1-endemic regions [237]. Critically, the genotoxic potency of AFB1 is substantially amplified under conditions of pre-existing hepatic oxidative stress: both hepatitis B viral infection and NAFLD-associated hepatic ROS reduce the activity of the nucleotide excision repair pathway responsible for AFB1-adduct removal, creating a synergistic interaction between dietary contaminant exposure and chronic oxidative hepatic injury in HCC initiation [238].

#### 4.3.3. Breast and Prostate Cancer

Dietary pro-oxidant exposure has been implicated in the pathogenesis of hormonally driven cancers—particularly breast and prostate cancer—through mechanisms that intersect with sex hormone metabolism and redox-sensitive signaling. In breast tissue, elevated ROS promote the cytochrome P450-mediated hydroxylation of estrogens to catechol estrogens (4-hydroxyestradiol), reactive quinone intermediates that form depurinating adducts with adenine and guanine bases, generating the apurinic sites and point mutations characteristic of breast cancer initiation [239]. Dietary saturated fats and obesity-associated adipose inflammation amplify this genotoxic pathway by increasing aromatase activity in breast adipose tissue, raising local estrogen concentrations and correspondingly increasing catechol estrogen–DNA adduct formation. High consumption of well-done meat and grilled foods—rich in PhIP and MeIQx—has been associated with increased breast cancer risk in several epidemiological studies, and PhIP has been shown to act as a mammary carcinogen in female rats through Nox-dependent ROS generation and ERα-dependent proliferative signaling [240].

In the prostate, oxidative stress contributes to carcinogenesis through epigenetic silencing of GSTP1 (encoding glutathione S-transferase Pi), the predominant antioxidant enzyme in a normal prostate epithelium; ROS-driven promoter hypermethylation of GSTP1 is the most common somatic epigenetic alteration in prostate cancer, occurring in greater than 90% of cases and detectable as an early event in prostatic intraepithelial neoplasia (PIN) [241]. The human prostate is uniquely vulnerable to dietary heterocyclic amine (HCA) carcinogens due to a fundamental imbalance in its metabolic detoxification machinery. Despite limited cytochrome P450-mediated N-hydroxylation capacity, the prostate efficiently converts N-hydroxy-PhIP into genotoxic N-acetoxy esters via an AcCoA-dependent pathway, with in vitro DNA adduct formation reaching up to 54 pmol/mg DNA [242]. GSTP1 constitutes the sole meaningful detoxification barrier against HCA-induced DNA adduct formation in the prostate, given the negligible contribution of glutathione S-transferase α 1 (GSTA1) to N-acetoxy-PhIP conjugation. Its near-universal epigenetic silencing via promoter hypermethylation in prostate adenocarcinomas consequently eliminates this protective capacity, leaving prostatic epithelial cells unguarded against dietary HCA genotoxicity.

Across cancer types, the dietary oxidative stress–cancer relationship is further contextualized by evidence from large-scale intervention trials, which have yielded instructive results: the Alpha-Tocopherol, Beta-Carotene Cancer Prevention (ATBC) Study demonstrated that β-carotene supplementation paradoxically increased lung cancer incidence in smokers, likely through pro-oxidant reactions of β-carotene oxidation products in the highly oxidized pulmonary environment of smokers [243]. This finding underscores a critical mechanistic principle: the protective versus pro-carcinogenic consequences of dietary antioxidant intake are context-dependent, determined by the prevailing tissue redox environment and the specific chemical form and dose of the antioxidant compound. Dietary antioxidant phytochemicals consumed as part of whole foods—embedded in a matrix of complementary phytochemicals, fiber, and micronutrients—consistently demonstrate cancer-preventive associations in prospective cohort studies, while high-dose isolated antioxidant supplements produce less consistent and sometimes adverse outcomes in clinical trials, reinforcing the biological superiority of whole-food dietary patterns over reductive nutrient supplementation strategies in cancer prevention [244].

### 4.4. Atherosclerosis

Atherosclerosis is the pathological foundation of the leading causes of cardiovascular mortality worldwide, encompassing coronary artery disease, myocardial infarction, and ischemic stroke. The disease is fundamentally an oxidative inflammatory process of the vascular wall in which chronic ROS exposure initiates, sustains, and amplifies the atherogenic cascade from endothelial dysfunction through foam-cell formation; plaque development; and, ultimately, plaque rupture [245]. Lipid peroxidation is now recognized as the earliest event in atherogenesis: ROS generated at the vascular wall oxidatively modify circulating low-density lipoprotein (LDL) particles—particularly the small, dense LDL (sdLDL) fraction characterized by high PUFA content and susceptibility to oxidation—to yield oxidized LDL (oxLDL), a potent pro-atherogenic ligand that drives virtually every step of the atherosclerotic process [246,247].

Major enzymatic sources of vascular ROS implicated in atherosclerosis include NADPH oxidases (Nox1, Nox2, and Nox5 in endothelial cells; Nox2 and Nox4 in macrophages), xanthine oxidase, uncoupled endothelial nitric oxide synthase (eNOS), and the mitochondrial ETC [248]. The central pathophysiological role of eNOS uncoupling in atherosclerosis deserves particular emphasis: under oxidative conditions, superoxide anion reacts with nitric oxide (NO) at near-diffusion-limited rates to form peroxynitrite (ONOO^−^), simultaneously depleting the vasodilatory and anti-inflammatory NO molecule and generating a highly reactive species that further oxidizes eNOS cofactor tetrahydrobiopterin (BH4), perpetuating eNOS uncoupling and superoxide generation in a self-amplifying cycle [248]. The resulting reduction in vascular NO bioavailability impairs endothelium-dependent vasodilation, promotes the expression of leukocyte adhesion molecules, and facilitates monocyte transmigration—the cellular hallmark of early atherosclerotic lesion formation [249].

Dietary pro-oxidant exposures directly impinge on each of these vascular pathways. Dietary saturated and trans fatty acids increase Nox2 activity and expression in endothelial cells; reduce NO bioavailability; and promote the production of small, dense, oxidation-susceptible LDL particles through mechanisms including PKC activation and impairment of LDL receptor-mediated clearance [19]. Dietary AGEs engage vascular RAGE to activate Nox1 and Nox2, augmenting endothelial and macrophage ROS production and accelerating the glycoxidation of LDL particles, rendering them particularly susceptible to macrophage scavenger receptor-mediated uptake and foam-cell formation [20]. Exogenous oxidized lipids from thermally processed foods—including 4-HNE, MDA, and lipid hydroperoxides—are absorbed from the gastrointestinal tract and exert systemic pro-oxidant effects; plasma MDA and F2-isoprostane concentrations increase postprandially following consumption of heated, oxidized fat-containing foods, and habitual consumption of diets with high oxidative lipid load is associated with elevated oxLDL and reduced paraoxonase-1 activity in epidemiological studies [9]. Conversely, habitual antioxidant nutrient intake—including vitamins C and E, polyphenols, and carotenoids—reduces circulating oxLDL, F2-isoprostanes, and inflammatory markers, consistent with a diet-mediated reduction in atherogenic oxidative burden [250].

It is important to note, however, that this relationship between dietary oxidative stress and atherosclerotic disease exhibits important species-specific variation: comparable dietary pro-oxidant exposures do not necessarily produce equivalent disease outcomes across species, and companion animals, particularly cats, rarely develop clinically significant atherosclerosis despite increasing rates of obesity and hypercholesterolemia. This notable exception, along with its underlying mechanistic basis, is discussed in detail in Section 4.5 in the context of the broader cross-species comparison of oxidative stress-associated chronic disease.

### 4.5. Veterinary Diseases

Companion animals—domestic dogs and cats—are afflicted by a spectrum of chronic diseases that mirrors, with striking fidelity, the oxidative stress-associated pathologies of their human counterparts. This convergence is not coincidental: as detailed in Section 1.4, companion animals share domestic environments with their owners; subsist almost exclusively on thermally processed commercial pet foods structurally analogous to ultra-processed human foods; and are consequently exposed to similar profiles of dietary AGEs, lipid oxidation products, acrylamide, and HCAs on a per-kilogram bodyweight basis. The veterinary literature has documented elevated systemic oxidative stress biomarkers in companion-animal diseases paralleling human conditions, though the mechanistic linkage to dietary pro-oxidant exposure remains less thoroughly characterized in the veterinary context than in human nutrition science.

#### 4.5.1. Canine and Feline Chronic Kidney Disease

Chronic kidney disease (CKD) is one of the most common and morbidity-associated conditions in aging dogs, estimated to affect 1–3% of the canine population at large, with substantially higher prevalence in geriatric cohorts [54]. It is likewise the leading metabolic disease of domestic cats, predominantly affecting animals older than 12 years; a large UK primary-care study of 353,448 cats reported an overall prevalence of 1.2% [251,252]. The pathophysiology of canine CKD shares essential features with its human counterpart: progressive nephron loss triggers compensatory hyperfiltration in surviving nephrons, generating a self-reinforcing cycle of intraglomerular hypertension, proteinuria, and tubular oxidative stress that accelerates irreversible glomerulosclerosis and tubulointerstitial fibrosis [253]. Elevated plasma isoprostanes, MDA, and protein carbonyls—along with reductions in erythrocyte SOD activity, plasma vitamin C, and total antioxidant capacity—have been documented in dogs with spontaneous CKD across multiple International Renal Interest Society (IRIS) staging categories, confirming that oxidative stress is a clinically relevant feature of canine renal disease progression [254]. Similarly, cats with CKD exhibit increased carbonyl stress and accumulation of carbonyl-derived end products, indicating that oxidative and carbonyl damage is also associated with feline renal disease [255].

In dogs, the dietary dimension of CKD-associated oxidative stress is particularly pertinent, given that processed commercial diets constitute the near-exclusive caloric source for most companion animals. High dietary phosphorus loads—characteristic of protein-rich extruded kibble—promote renal hyperphosphatemia that activates fibroblast growth factor 23 (FGF23) signaling, contributing to vascular and renal oxidative stress [256]. The same axis operates in cats, in which excessive intake of highly available dietary phosphorus increases gastrointestinal absorption and renal excretion, while FGF23- and parathyroid hormone-mediated reductions in tubular phosphorus reabsorption raise the intratubular phosphorus load, contributing to glucosuria, microalbuminuria, and reduced creatinine clearance [257]. Heat-processed commercial diets further deliver dietary AGEs (CML and CEL) alongside protein oxidation products generated during extrusion—including protein carbonyls and Amadori rearrangement products—which are absorbed from the gastrointestinal tract and excreted renally, imposing an added carbonyl and pro-oxidant burden on the tubular cells responsible for amino acid reabsorption [258]. Domestic cats additionally exhibit renal accumulation of pro-oxidant trace elements, together with relatively low concentrations of antioxidant minerals such as copper and zinc, which may weaken SOD-dependent antioxidant defenses and promote ROS-mediated tubular injury, chronic interstitial nephritis, and fibrosis, although a direct causal relationship with feline CKD has not been established [259]. Given the analogous pathophysiology of CKD across species, the AGE–RAGE–sRAGE axis observed in human CKD patients—where oxidative stress and impaired renal clearance drive AGE accumulation, NF-κB-mediated inflammation, and disease progression—is presumed to play a comparable role in dogs with spontaneous CKD [128].

Consistent with this canine and feline evidence, chronic kidney disease provides a robust cross-species parallel. In both humans and dogs, CKD progression is driven by a cycle of hyperfiltration-induced oxidative stress, ROS-mediated glomerular and tubulointerstitial injury, progressive nephron loss, and further compensatory hyperfiltration [253]. Therapeutic responsiveness to dietary protein and phosphorus restriction and omega-3 supplementation is demonstrated in both species, and the oxidative stress biomarker profiles—elevated isoprostanes, MDA, and protein carbonyls with reduced antioxidant enzyme activities—are qualitatively similar [129,260]. This convergence supports the use of dietary oxidative stress as a therapeutically modifiable disease driver in both clinical human nephrology and veterinary internal medicine.

#### 4.5.2. Inflammatory Bowel Disease

Canine inflammatory bowel disease (IBD) is a complex, immunologically mediated gastrointestinal disorder characterized by chronic infiltration of inflammatory cells into the mucosa of the stomach, small intestine, or colon, resulting in disrupted nutrient absorption; protein-losing enteropathy; and clinical signs including chronic vomiting, diarrhea, and weight loss [261]. Its pathogenesis involves intricate interactions among genetic predisposition, mucosal immune dysregulation, intestinal microbiota dysbiosis, and environmental factors—with diet constituting a dominant environmental determinant [262]. Elevated serum biomarkers of oxidative stress, including MDA and oxidized protein species, have been documented in dogs with idiopathic IBD relative to healthy controls [254], and a sustained increase in biogenic amine putrescine—an indicator of persistent oxidative stress—has been identified as a metabolomic feature of inadequately treated canine IBD [263].

Dietary processing-derived additives and contaminants are recognized contributors to mucosal oxidative stress in canine IBD. Commercial pet-food additives—including certain emulsifiers (carboxymethylcellulose and polysorbate 80), artificial colorants, and preservatives—have been shown to disrupt intestinal-barrier integrity, increase mucosal permeability to luminal antigens and LPS, and promote low-grade mucosal inflammation and ROS generation through pattern-recognition receptor activation [264]. Dysbiosis of the intestinal microbiota, a consistent feature of canine IBD involving reduced abundances of short-chain fatty acid (SCFA)-producing *Lachnospiraceae* and *Ruminococcaceae* alongside increased Enterobacteriaceae, impairs intestinal antioxidant defenses by reducing butyrate production—the primary luminal antioxidant substrate for colonocytes—and alters bile acid metabolism, reducing the availability of secondary bile acids with antioxidant and anti-inflammatory properties [265]. Dietary interventions—particularly hydrolyzed protein diets supplemented with prebiotics and glycosaminoglycans (GAGs)—have been shown to favorably modulate the serum metabolomic profile of IBD dogs, increasing taurine (a mucosal antioxidant and membrane-stabilizing amino acid) and restoring lipid metabolic balance, consistent with a partial correction of oxidative mucosal pathology through nutritional intervention [266].

In cats, consumption of thermally processed commercial diets increases dietary exposure to advanced glycation end products (AGEs), including CML and CEL. The greater urinary excretion of these AGEs in cats fed processed diets suggests gastrointestinal absorption of at least a portion of dietary AGEs [258,267]. Once absorbed, dietary AGEs may engage intestinal RAGE-associated signaling, which is associated with increased oxidative stress, as reflected by elevated lipid peroxidation markers such as MDA, together with enhanced iNOS/NO signaling and endothelial activation. Increased endothelial expression of adhesion molecules such as ICAM-1 can facilitate leukocyte recruitment into intestinal tissues, and RAGE signaling further promotes inflammatory mediators, including IL-1β. Accompanied by epithelial injury and intestinal lesions, these oxidative and inflammatory responses may aggravate intestinal inflammation [268]. Separately, feline diets rich in oxidation-prone polyunsaturated fatty acids are susceptible to lipid oxidation during processing and storage, leading to the accumulation of lipid hydroperoxides and secondary oxidation products such as MDA [88,269]. When glutathione peroxidase 4 (GPX4)-dependent detoxification of lipid hydroperoxides is insufficient, lipid ROS and phospholipid peroxidation can accumulate in colonic fibroblasts [270]. This lipid-peroxidation-prone fibroblast state can promote pathogenic stromal-epithelial cross-talk, increasing epithelial susceptibility to ferroptosis and cell death. The resulting epithelial and barrier injury is associated with greater intestinal tissue damage and inflammatory-cell infiltration, ultimately exacerbating colitis and potentially contributing to IBD progression [88,270].

#### 4.5.3. Pet Obesity and Associated Oxidative Comorbidities

Obesity is now the most prevalent nutritional disease in companion animals, with an estimated 30–40% of dogs and cats in developed countries classified as overweight or obese—a prevalence strikingly similar to that observed in human populations of the same geographic regions [271,272]. This epidemiological parallel is biologically meaningful: companion animals and their owners share not only living environments but also dietary habits, including consumption of energy-dense, processed, and frequently offered calorie-rich foods and treats [273].

The most compelling cross-species parallel is the shared burden of obesity-related metabolic disease. In humans, dogs, and cats alike, the combination of energy-dense processed food consumption and physical inactivity drives adipose tissue expansion, adipokine dysregulation, systemic low-grade inflammation, and oxidative stress-mediated insulin resistance [272,274]. The mechanisms—elevated circulating non-esterified fatty acids (NEFAs) promoting mitochondrial ROS, tumor necrosis factor-α (TNF-α)-mediated IRS-1 serine phosphorylation, and reduced adiponectin signaling—are conserved across these species, and the downstream metabolic consequences (T2DM in humans and cats; insulin resistance, orthopedic disease, and reduced longevity in dogs) follow comparable pathophysiological logic [275]. Notably, a 14-year controlled feeding study in Labrador retrievers demonstrated that dogs maintained at ideal body weight lived an average of 1.8 years longer than their ad libitum-fed obese littermates, with delayed onset of chronic diseases, underscoring the lifespan consequences of dietary energy balance and its attendant oxidative stress burden in dogs as in humans [271].

As in humans, adipose tissue in obese companion animals functions as an active endocrine organ rather than a passive energy reservoir, secreting an altered adipokine profile characterized by elevated leptin, TNF-α, interleukin-6 (IL-6), and C-reactive protein alongside reduced adiponectin, establishing a state of chronic low-grade systemic inflammation and oxidative stress [275]. In obese dogs, expanded visceral adipose tissue drives excessive mitochondrial β-oxidation of circulating NEFAs, generating ROS that overwhelm cellular antioxidant defenses, promote lipotoxicity in non-adipose tissues, and induce insulin resistance through JNK- and IKKβ-mediated IRS-1 serine phosphorylation [276]. Elevated plasma MDA, protein carbonyl, and isoprostane concentrations have been documented in obese dogs relative to lean controls, confirming that systemic oxidative stress is a measurable feature of canine obesity [277].

In cats, obesity is the leading risk factor for the development of type 2 diabetes mellitus, with an approximately four-fold increased risk in obese compared to lean individuals [275]. Feline obesity is associated with adipokine dysregulation—elevated leptin and reduced adiponectin—paralleling the human metabolic syndrome, and leptin-driven pro-inflammatory signaling increases tissue ROS production and reduces insulin receptor sensitivity through mechanisms conserved across mammalian species [278]. The high-carbohydrate, high-fat formulation of many commercial extruded pet foods, combined with the sedentary indoor lifestyle of companion animals, creates a dietary and environmental context that structurally parallels the conditions driving the human obesity epidemic, with oxidative stress serving as a shared pathogenic bridge between energy excess and metabolic disease across species [272]. The chronic consumption of processed pet foods with inadequate antioxidant supplementation—particularly those containing rendered animal fats susceptible to lipid oxidation during storage—may therefore contribute to the oxidative burden underlying obesity-associated comorbidities in companion animals, though controlled dietary intervention studies establishing this causal chain in vivo remain limited.

#### 4.5.4. Cancer in Companion Animals and Livestock: Dietary ROS as a Shared Carcinogenic Driver

Cancer represents a leading cause of mortality in companion animals and a significant source of economic loss in livestock production, and accumulating evidence implicates dietary pro-oxidant exposure as a shared carcinogenic driver across veterinary species. In dogs, oxidative stress has been mechanistically linked to spontaneous tumor development through ROS-mediated DNA damage, epigenetic silencing of tumor-suppressor loci, and redox-driven activation of oncogenic signaling pathways conserved between canine and human cancers [279]. Serum MDA concentrations—a validated marker of lipid peroxidation-derived oxidative burden—are significantly elevated in cancer-bearing dogs relative to healthy controls across multiple tumor types, including lymphoma, mammary carcinoma, and mast cell tumors, establishing oxidative stress as a measurable systemic feature of canine malignancy [279]. The dietary contribution to this oxidative burden is mechanistically grounded in the documented presence of HCAs (PhIP and MeIQx) and lipid oxidation products in commercial extruded pet foods at concentrations approaching those in thermally processed human foods, implicating chronic dietary pro-oxidant exposure as a plausible upstream contributor to spontaneous canine carcinogenesis in a manner directly analogous to the dietary HCA–cancer axis characterized in human epidemiology [176].

In livestock, dietary AFB1—generated by Aspergillus species contaminating improperly stored feed commodities—constitutes the most potent and mechanistically characterized dietary hepatocarcinogen in veterinary medicine: CYP3A4-mediated epoxidation of AFB1 generates a reactive 8,9-exo-epoxide that forms mutagenic N7-guanine DNA adducts, and the intracellular ROS produced during AFB1 metabolism simultaneously impair nucleotide excision repair, amplifying the genotoxic consequence of each formed adduct and rendering no animal model exposed to AFB1 free from hepatocellular carcinoma development [237]. The convergence of dietary ROS-mediated carcinogenesis across companion animals and livestock—operating through mechanisms shared with human cancer biology—positions veterinary oncology as both a translational model and an independent domain of clinical relevance within the broader framework of diet-induced oxidative pathology.

## 5. Comparative Perspective: Humans vs. Companion Animals

The central novelty of the present review resides in its dual-species analytical framework. While the preceding sections have described oxidative stress-associated diseases in humans and companion animals in parallel, this section undertakes an explicit comparative synthesis of the dietary exposure conditions, metabolic processing characteristics, and disease susceptibility profiles of humans and companion animals, with the specific aim of identifying points of convergence that support translational insights and points of divergence that must be accounted for when extrapolating mechanistic or epidemiological findings across species.

### 5.1. Dietary Exposure: Parallels and Divergences in Pro-Oxidant Burden

The most fundamental point of convergence between humans and companion animals is their shared reliance on thermally processed foods as the primary caloric source (Section 1.3 and Section 1.4). Because human ultra-processed foods and commercial pet foods are manufactured under broadly analogous high-temperature conditions, they carry structurally similar processing-induced contaminant profiles: Maillard reaction-derived AGEs, acrylamide, HCAs, PAHs, lipid peroxidation products (4-HNE and MDA), and 3-MCPD/glycidyl esters, as detailed in Section 2.2 [36,49,193]. This structural similarity in processing conditions implies that the qualitative spectrum of dietary pro-oxidant exposure is broadly comparable across species. This parallel is not merely inferential: PhIP, MeIQx, and selected PAHs have been detected in commercial pet foods at per-kilogram concentrations approaching those of thermally processed human foods [176].

Critical quantitative divergences in dietary pro-oxidant exposure nonetheless exist. The fat content of commercial pet foods—particularly dry kibble for dogs—is substantially higher on a caloric basis than most human dietary patterns, often comprising 25–40% of metabolizable energy (ME), and the rendered animal-fat fractions incorporated into pet foods as palatability enhancers are particularly susceptible to lipid oxidation during manufacturing and post-opening storage [51]. Consequently, companion animals may receive a higher dietary burden of secondary lipid oxidation products (4-HNE, MDA, and lipid hydroperoxides) per kilogram of body weight than humans consuming typical mixed diets, a disparity that could compound oxidative stress accumulation over the lifespan. In contrast, humans are exposed to dietary acrylamide at particularly high levels through coffee, fried potatoes, and baked goods—food categories without direct equivalents in companion animal diets—though extrusion-processed pet-food kibble has been documented to contain measurable acrylamide concentrations [280]. The diversity of human dietary exposure—spanning raw, lightly processed, and heavily processed food items depending on individual dietary choices—introduces a degree of inter-individual variability in pro-oxidant burden that is largely absent in companion animals, whose diets are typically formulated to a fixed recipe with minimal meal-to-meal variation.

An additional exposure dimension unique to companion animals is the frequency of long-term, exclusive consumption of a single commercial diet formulation—sometimes spanning the entire lifespan of the animal. This dietary monotony has no human equivalent and raises the possibility of cumulative pro-oxidant burden amplification from chronic exposure to any contaminants present in a given formulation, as well as potential depletion of dietary antioxidant reserves not adequately compensated for by formulation antioxidants that may degrade during storage. The observation that commercial pet-food antioxidant supplementation (commonly tocopherol-based) is formulated to meet minimum Association of American Feed Control Officials (AAFCO) requirements at the time of manufacture but may be substantially depleted by the time of consumption—due to oxidative degradation during storage, particularly after bag opening—represents a clinically relevant nutritional vulnerability in companion animals that has no straightforward human parallel [51].

### 5.2. Gastrointestinal Absorption: Species-Specific Determinants of Dietary Pro-Oxidant Uptake and Oxidative Burden

While Section 5.1 established that the qualitative spectrum of dietary pro-oxidants presented to humans and companion animals is broadly comparable, the biological consequence of any ingested compound depends not on its luminal concentration but on the fraction that is actually absorbed across the intestinal epithelium and delivered to systemic circulation—a step that diverges markedly across species and thereby shapes the resulting oxidative burden.

The secondary lipid oxidation products (4-HNE, MDA, and lipid hydroperoxides) abundant in the high-fat rendered fractions of commercial pet food are not confined to the lumen but can enter the body, albeit in a form-dependent manner. Dietary triacylglycerol and linoleic-acid hydroperoxides are largely decomposed to aldehydes within the stomach rather than being absorbed intact, and at dietarily relevant doses, the parent hydroperoxides do not reach the intestine so that it is chiefly their decomposition products that become available for uptake [281]. Peroxidized lipids and their aldehydic breakdown products are nonetheless absorbed and transported via the intestinal lymphatics, a process constrained by mucosal glutathione such that depletion of mucosal GSH increases their lymphatic transport [282], and in humans, a red-meat meal produces a rapid postprandial rise in plasma malondialdehyde, an accumulation attenuated when dietary polyphenols suppress lipid peroxidation in the gastric compartment—direct evidence that meal-derived MDA is absorbed into circulation [283]. Uptake has likewise been demonstrated for individual reactive aldehydes: heme-iron-driven lipid peroxidation in the intestinal lumen of rats generated 4-HNE, whose major urinary metabolite rose measurably, indicating absorption, and separately administered stable isotope-labeled 4-HNE reached extra-intestinal tissues in a bioactive form, forming HNE–protein adducts in the liver, heart, and skeletal muscle [284].

Heterocyclic amines such as PhIP are absorbed only to a limited extent in the small intestine [284], with a substantial fraction of the unmetabolized compound escaping absorption and reaching the colon, where the distal colon may even actively secrete PhIP back into the lumen [285]. Crucially, the enterocyte is not a passive conduit but a first metabolic filter: the intestinal mucosa metabolizes xenobiotics through both phase I and phase II reactions and can significantly reduce the oral bioavailability of substrate compounds before they reach the portal circulation [286]. This filter is double-edged, however, as first-pass metabolism can either detoxify a compound through phase II conjugation to water-soluble, readily excreted derivatives or bioactivate it, as when phase I oxidation converts a relatively inert pro-carcinogen into a reactive, DNA-binding intermediate; the net consequence therefore depends on the substrate and on which enzymatic route predominates. Because the efficiency of this filter is, itself, species-divergent, the pronounced phase II conjugation deficiencies of the cat (detailed in Section 5.3) chiefly impair the detoxifying arm, curtailing the conjugation and clearance of reactive intermediates and thereby prolonging tissue exposure to activated metabolites rather than simply lowering their systemic availability.

These absorptive differences are further modulated by gross gut architecture and kinetics: as carnivores, dogs and cats possess a relatively shorter digestive tract than omnivorous mammals, which favors reliance on highly digestible diets [286], while total gastrointestinal transit time differs significantly between the two species under comparable feeding conditions [287], together determining the residence time over which luminal pro-oxidants are absorbed, activated, or delivered intact to the portal circulation. That dietary oxidized lipids exert biologically meaningful effects in the animal intestine is well established in production species: broilers fed peroxidized soybean oil show dose-dependent increases in jejunal and plasma malondialdehyde, declines in mucosal and systemic antioxidant capacity, and suppression of intestinal secretory IgA and CD4/CD8 populations [288], and more recent work in piglets has extended these observations across multiple intestinal segments, linking peroxidized lipid intake to segment-specific oxidative stress, altered intestinal morphology and inflammatory signaling, and shifts in the gut microbiome [289]. It must be emphasized, however, that the intestinal absorption and handling of these dietary pro-oxidants has been directly characterized almost exclusively in rodent and human models; in companion animals and, indeed, in most non-rodent species, comparable quantitative absorption data are essentially absent, and the livestock evidence, itself, is largely indirect—demonstrating that dietary oxidized lipids provoke intestinal oxidative stress and mucosal injury rather than quantifying their uptake. The interspecies differences inferred here therefore rest on divergences in gut architecture, transit, and mucosal enzyme complement rather than on direct measurement of pro-oxidant uptake in companion animals, constituting a substantive gap in comparative nutritional toxicology and a priority for future investigation. Thus, equivalent dietary pro-oxidant exposure cannot be assumed to translate into an equivalent systemic oxidative dose across species, and this absorptive divergence must be accounted for before the metabolic differences and disease associations that follow can be interpreted.

### 5.3. Metabolism Differences: Species-Specific Toxicokinetic Determinants of Dietary Oxidant Susceptibility

While the dietary pro-oxidant exposure profiles of companion animals and humans are broadly similar, substantial interspecies differences in metabolic processing of dietary oxidants fundamentally alter the biological consequences of equivalent exposures. These toxicokinetic divergences operate at the levels of bioactivation, conjugation, and elimination and have direct implications for tissue-level oxidative damage and organ-specific disease susceptibility.

The CYP enzyme system, which mediates the bioactivation of HCAs, PAHs, acrylamide, and numerous other dietary pro-oxidants, exhibits significant interspecies variation in expression, catalytic activity, and inducibility. In humans, CYP1A2 is the primary phase I enzyme responsible for HCA N-hydroxylation, converting dietary PhIP and MeIQx into reactive intermediates that are subsequently O-acetylated by NAT2 to form mutagenic DNA adducts in target epithelia. CYP1A2 activity varies more than 40-fold across individuals and is strongly inducible by high-HCA diets, smoking, and cruciferous vegetable intake, while the *NAT2* slow/rapid acetylator polymorphism further modulates individual cancer risk—with the rapid acetylator phenotype conferring significantly elevated colorectal cancer risk in the context of high red-meat consumption. Dogs possess hepatic CYP1A2 isoforms capable of HCA N-hydroxylation and exhibit urinary excretion of HCA metabolites following consumption of a processed diet, confirming that bioactivation pathways relevant to oxidative and genotoxic damage are operative in the canine species [191]. However, canine *CYP1A2* harbors a non-synonymous single-nucleotide polymorphism that substantially reduces its catalytic efficiency relative to the human enzyme, and this polymorphism is segregating in canine populations, creating inter-individual variation in HCA bioactivation efficiency within dogs that parallels but is distinct from the slow/rapid acetylator pharmacogenomic variation in humans [290]. Cats present an even more pronounced metabolic divergence: they are deficient in hepatic glucuronyl transferase activity and possess reduced sulfotransferase capacity, severely impairing the phase II conjugation of activated HCA metabolites and phenolic compounds. This deficiency, evolutionarily explained by the obligate carnivory of *Felis catus* in an environment with limited plant xenobiotic exposure, paradoxically renders cats more vulnerable to prolonged tissue exposure to reactive HCA intermediates generated by a CYP system that remains functional [290].

Acrylamide metabolism provides another instructive comparative example. In all three species, acrylamide is epoxidized by CYP2E1 to glycidamide, the more reactive genotoxic metabolite, and both acrylamide and glycidamide form hemoglobin adducts that serve as biomarkers of exposure. In humans, CYP2E1 is strongly inducible by fasting, ethanol, obesity, and ketogenic diets—conditions that substantially increase endogenous CYP2E1 activity and, consequently, acrylamide bioactivation from dietary sources [291]; the co-occurrence of obesity and alcohol consumption acts synergistically on CYP2E1 induction, potentially amplifying the genotoxic burden of dietary acrylamide in metabolically vulnerable subpopulations. The extent to which analogous metabolic inducibility of canine and feline CYP2E1 occurs under typical pet-kept conditions remains poorly characterized, representing a gap in comparative toxicokinetics with direct relevance to dietary acrylamide risk assessment in companion animals. Feline deficiency in glutathione S-transferase (GST)-mediated conjugation of acrylamide and glycidamide further extends the potential for oxidative tissue damage from these dietary contaminants relative to dogs or humans [290].

Endogenous antioxidant metabolism also diverges meaningfully between species. Cats cannot synthesize taurine or arachidonic acid de novo, requiring dietary provision of these nutrients, with taurine deficiency documented in cats fed certain commercial diets, particularly those based on plant proteins [292]. Taurine is both a direct antioxidant and a membrane stabilizer with mucosal protective functions, and its deficiency amplifies susceptibility to ROS-mediated organ damage. In contrast to humans—in whom the *GULO* gene encoding L-gulonolactone oxidase has been rendered non-functional through accumulated mutations, imposing an absolute dietary requirement for vitamin C—cats retain functional endogenous ascorbate biosynthesis. Nevertheless, this synthetic capacity may be insufficient to offset the elevated oxidative burden imposed by thermally processed, antioxidant-depleted diets so that dietary vitamin C can still contribute meaningfully to antioxidant replenishment under conditions of chronic oxidative stress [293]. This shared dependency implies that diets high in thermally processed foods with low antioxidant content impose a comparable oxidative vulnerability in both humans and cats—a notable point of biological convergence that is rarely acknowledged in either clinical nutrition or veterinary practice. Dogs synthesize ascorbate hepatically, and the benefit of supplemental vitamin C in dogs appears context-dependent: in healthy dogs already replete in vitamin E, supplementation produced no clear improvement in antioxidative capacity and only marginal immunological changes, whereas clinical benefits are more plausible under conditions of elevated oxidative demand [294].

### 5.4. Cross-Species Parallels and Divergences in Oxidative Stress-Associated Disease

The comparative disease profile across humans and companion animals reveals both remarkable parallels that support a shared dietary oxidative stress etiology and instructive divergences that illuminate species-specific disease modifiers. Table 3 provides a structured overview of these cross-species disease comparisons.

Taken together, the comparative analysis of dietary ROS exposure, metabolic processing, and disease susceptibility in humans and companion animals supports three overarching conclusions. First, the structural similarity of thermally processed human foods and commercial pet foods generates a qualitatively similar spectrum of dietary pro-oxidant exposure across species, establishing a biological foundation for cross-species translational relevance. Second, interspecies differences in toxicokinetic processing—as detailed in Section 5.3—modulate the biological consequences of equivalent dietary exposures, creating species-specific windows of vulnerability that must be considered in comparative risk assessment. Third, the remarkable prevalence of shared oxidative stress-associated diseases such as metabolic syndrome, obesity, CKD, IBD, and diet-associated cancer across humans and their companion animals living in shared domestic environments provides epidemiological support for the role of shared dietary pro-oxidant exposures in driving parallel chronic disease burdens and simultaneously positions companion animals as a uniquely valuable naturalistic translational model for investigating diet-induced oxidative pathology in a setting that approximates human environmental conditions more closely than any rodent laboratory model.

From a comparative pathology perspective, these mechanisms appear broadly conserved across species, including humans, laboratory rodents, and other mammals, suggesting that insights from experimental models are directly relevant to both human and veterinary health. Given the increasing reliance on highly processed foods in human diets and thermally processed feeds in companion animals and livestock, processing-induced toxic compounds and their ROS-mediated mechanisms represent an important but often under-recognized, contributor to the initiation and progression of ROS-associated diseases [136] (Table 4). Moreover, nutritional deficiencies in antioxidant vitamins, trace elements, or endogenous antioxidant precursors are likely to exacerbate susceptibility to these toxicants, lowering the threshold at which processing-induced compounds can disrupt redox homeostasis and precipitate pathology.

## 6. Dual Redox Roles of Dietary Compounds: Context-Dependent Pro-Oxidant and Antioxidant Activities

Dietary compounds that interact with cellular redox networks have historically been framed as antioxidants—agents that counteract food-derived ROS through transcriptional reprogramming, direct radical interception, membrane remodeling, and mitochondrial bioenergetics. However, a mechanistically rigorous examination reveals that these same compounds are electrophilic, redox-active molecules capable of generating oxidative stress. Concentration, cellular redox context, metal ion availability, and species-specific metabolic capacity collectively determine whether a given dietary compound functions as a pro-oxidant stressor, an adaptive signal, or a cytoprotective agent. Framed in these terms, many dietary redox-active compounds act not by neutralizing ROS but by imposing a transient, low-level oxidative challenge within the eustress range, eliciting a hormetic adaptive response [298,299]. This section reinterprets the four canonical nutritional redox pathways through the lens of this dual redox nature, emphasizing that induction of antioxidant gene expression is, itself, evidence of prior or concurrent oxidative stress imposed by these compounds.

### 6.1. Dietary Polyphenols and Nrf2/ARE Activation: Evidence of Electrophilic Stress

Curcumin exemplifies the dual redox character of dietary polyphenols with precision: its α,β-unsaturated carbonyl moiety undergoes Michael addition with Keap1 cysteine residues [300]—a reaction chemically indistinguishable from the cysteine adduction caused by lipid peroxidation products such as 4-hydroxynonenal (4-HNE). At concentrations achievable in the hepatic portal circulation following dietary intake, curcumin generates measurable intracellular ROS via redox cycling and copper-catalyzed autoxidation, and its pro-oxidant activity has been demonstrated to precede—and mechanistically drive—subsequent Nrf2 nuclear translocation in hepatocytes, colonocytes, and vascular endothelial cells.

Sulforaphane carbamylates Keap1 C151 with high efficiency [213,300]; this modification is the molecular signature of an isothiocyanate electrophile reacting with cellular nucleophiles—precisely the class of reactivity that defines toxic electrophilic species. Resveratrol activates SIRT1-mediated Nrf2 deacetylation to prolong nuclear retention [301] but also generates superoxide via mitochondrial Complex I inhibition at concentrations exceeding 25 μM. Epigallocatechin-3-gallate (EGCG) modifies Keap1 through both direct adduction and PI3K/Akt-GSK3β pathway modulation [107] while simultaneously producing extracellular H_2_O_2_ via auto-oxidation of its galloyl moiety at rates sufficient to induce DNA strand breaks in cultured cells at physiologically plausible concentrations [302]. Quercetin inhibits the Keap1-Nrf2 protein–protein interaction through Kelch-domain binding [104] while undergoing transition-metal-catalyzed oxidation to semiquinone radicals and o-quinones that form protein adducts analogous to those of endogenous lipid peroxidation products.

The downstream transcriptional program induced by these compounds—encompassing GCL, GSTs, HO-1, NQO1, TrxR, and ferritin-should therefore be interpreted as an adaptive stress response to polyphenol-imposed oxidative and electrophilic injury rather than a direct antioxidant mechanism [198]. Species differences in phase II metabolism and GSH biosynthetic capacity modulate the threshold between adaptive hormesis and cytotoxic pro-oxidant injury [199,200].

### 6.2. Dietary Polyphenols as Context-Dependent Radical Scavengers and Pro-Oxidant Metal Redox Catalysts

The direct radical-intercepting properties of dietary polyphenols are mechanistically well established: phenolic hydroxyl groups donate hydrogen atoms to lipid peroxyl radicals (LOO•) via hydrogen-atom transfer (HAT) and single-electron transfer (SET) mechanisms, forming resonance-stabilized phenoxyl radicals that are insufficiently reactive to propagate lipid peroxidation chain reactions [303]. The thermodynamic basis of this activity resides in the O-H bond dissociation enthalpy (BDE): quercetin, EGCG, and curcumin exhibit BDE values thermodynamically favorable for radical quenching [67]. However, the same electronic properties that lower the O-H BDE and facilitate radical donation also render the resulting phenoxyl radicals capable of reducing molecular oxygen to superoxide (O_2_•^−^) under aerobic conditions, particularly in the presence of transition metals.

Catechol and galloyl moieties present in tea catechins and ellagitannins chelate redox-active Fe^2+^ and Cu^+^ through stable metal–polyphenol coordination complexes, suppressing Fenton-type hydroxyl radical generation catalyzed by dietary heme iron [303]. Critically, however, polyphenol–metal complexes are redox-active, and the Fe^3+^/Fe^2+^ cycling facilitated by polyphenol ligands under reducing conditions can accelerate rather than suppress Fenton chemistry when the reducing equivalents supplied by polyphenol autoxidation maintain iron in the catalytically active ferrous state [304]. The net outcome—pro-oxidant or antioxidant—depends on the molar ratio of polyphenol to iron; the local pH; and the presence of co-substrates such as ascorbic acid, which regenerates Fe^2+^ from Fe^3+–^polyphenol complexes [305]. In companion-animal diets combining plant-derived polyphenols with heme-rich meat ingredients, the coincidence of polyphenol autoxidation products, dietary heme iron, and variable gastric pH may shift the polyphenol redox balance decisively toward pro-oxidant activity.

### 6.3. Omega-3 Polyunsaturated Fatty Acids: Membrane Redox Signaling and Peroxidation Vulnerability

Long-chain omega-3 PUFAs—principally eicosapentaenoic acid (EPA, 20:5(n−3)) and docosahexaenoic acid (DHA, 22:6(n−3))—modulate cellular redox biology through membrane phospholipid remodeling, eicosanoid biosynthesis, and transcriptional regulation. Incorporation of EPA and DHA into phospholipid bilayers competitively displaces arachidonic acid (AA, 20:4n−6) from the sn-2 position of glycerophospholipids, reducing substrate availability for cyclooxygenase-2 (COX-2) and 5-lipoxygenase (5-LOX)-mediated generation of pro-inflammatory eicosanoids, such as prostaglandin E_2_ (PGE_2_) and leukotriene B_4_ (LTB_4_), that activate NADPH oxidase through PKC-dependent phosphorylation of the p47phox regulatory subunit [306]. EPA and DHA also serve as precursors for specialized pro-resolving mediators (SPMs)—resolvins, protectins, and maresins—which inhibit neutrophil NADPH oxidase activation and upregulate HO-1 via Nrf2 [307].

The high degree of unsaturation that confers EPA and DHA with signaling versatility also renders them among the most peroxidation-susceptible biomolecules in the mammalian membrane lipidome: the peroxidizability index of DHA (~320) is approximately 13-fold greater than that of oleic acid (~25), reflecting the five additional bis-allylic methylene positions available for hydrogen abstraction. Under conditions of elevated ROS generation, n−3 PUFA-enriched membranes become preferential substrates for lipid peroxidation, generating cytotoxic aldehydic products including 4-HHE from EPA/DHA oxidation—a structural analog of the 4-HNE derived from arachidonate peroxidation [308]. At concentrations generated by membrane peroxidation, 4-HHE forms protein carbonyl adducts, impairs mitochondrial Complex I activity, and activates pro-apoptotic signaling cascades. The paradoxical observation that n−3 PUFA-enriched membranes display enhanced Nrf2 induction is therefore most parsimoniously explained by 4-HHE-mediated Keap1 cysteine modification (see Section 3.6), not by an intrinsic antioxidant property of the fatty acids themselves.

In companion animals, this pro-oxidant dimension is compounded by species-specific metabolic constraints. The markedly limited Δ6-desaturase activity documented in cats creates an absolute dependency on preformed dietary DHA while simultaneously reducing the capacity to upregulate desaturation in response to elevated membrane peroxidative stress [51,309]. Dietary omega-3 supplementation in cats must therefore be contextualized against the antioxidant status of the diet as a whole: supplementation in the absence of adequate vitamin E—the principal chain-breaking antioxidant for membrane PUFA peroxidation—may amplify rather than attenuate oxidative membrane injury [310].

### 6.4. Dietary Modulation of Mitochondrial Redox Homeostasis: Adaptive Responses to Metabolic Pro-Oxidant Signaling

Caloric restriction and its molecular mimetics—including resveratrol and NAD^+^-boosting compounds such as nicotinamide riboside (NR) and nicotinamide mononucleotide (NMN)—activate the SIRT1/PGC-1α transcriptional axis, upregulating mitochondrial biogenesis genes, including nuclear respiratory factor 1 (*NRF1*) and mitochondrial transcription factor A (*TFAM*), and inducing mitochondrial antioxidant enzymes including MnSOD (SOD2) and mitochondrial thioredoxin 2 (TRX2) [311]. However, the induction of mitochondrial antioxidant capacity by these compounds is, itself, a homeostatic response to metabolic stress signals generated by the compounds themselves. Resveratrol inhibits mitochondrial Complex I at pharmacological concentrations, transiently increasing superoxide generation at the Qo site of Complex III; the resulting mitochondrial ROS signal activates AMPK and SIRT1, initiating the biogenesis response that ultimately reduces ROS output [301]. Resveratrol’s mitochondrial protection is therefore not an intrinsic antioxidant property but a conditional outcome: it occurs only when the cell possesses sufficient adaptive capacity to respond to resveratrol’s own pro-oxidant insult.

Dietary coenzyme Q10 (CoQ10) functions as either a mitochondrial antioxidant or a pro-oxidant source, depending on the integrity of the electron transport chain into which it is introduced. Under normal ETC function, CoQ10 acts as an obligate electron carrier between Complexes I/II and III, and its reduced form (ubiquinol) quenches lipid peroxyl radicals within the inner mitochondrial membrane [312]. Dietary supplementation increases the ubiquinol: ubiquinone ratio, shifting the semiquinone radical equilibrium toward the fully reduced form and reducing single-electron oxygen reduction at the Qo site—a well-established antioxidant mechanism at physiological doses.

However, the same semiquinone radical intermediate generated during CoQ10 cycling is, itself, an ROS-producing species. When ETC flux is impaired—as occurs during ischemia–reperfusion, inhibition of ETC complexes by dietary toxins, or mitochondrial dysfunction associated with aging—increased ubiquinone substrate availability paradoxically amplifies Qo-site superoxide generation rather than suppressing it. The conditions under which CoQ10 is most commonly supplemented—cardiovascular disease, aging, and mitochondrial myopathy—are precisely those under which ETC function is most likely to be compromised. In dogs, dietary CoQ10 reduces mitochondrial ROS generation and preserves ETC-complex activity in aging cardiac tissue [313]; however, this benefit presupposes sufficient residual ETC integrity to sustain the antioxidant cycling mode. Where that integrity is absent, the pro-oxidant outcome predominates.

## 7. Knowledge Gaps and Future Research Directions

The mechanistic and comparative framework developed in the preceding sections reveals not only what is known about dietary ROS-mediated pathology across species but also the contours of what remains unresolved. Five priority knowledge gaps emerge directly from the reviewed evidence, each representing a point at which the current literature is insufficient to support the quantitative, species-specific, or translational conclusions that would be required for evidence-based clinical and regulatory application. These gaps are not independent: they share common methodological limitations and, if addressed in concert, would substantially advance the field.

### 7.1. Absence of Long-Term, Species-Specific Dose–Response Data for Chronic Dietary Pro-Oxidant Exposure

As documented throughout Section 2 and Section 3, the pro-oxidant compounds generated during thermal food processing—AGEs, acrylamide, HCAs, PAHs, lipid oxidation products, N-nitroso compounds, and preservative-associated oxidants—have been individually characterized with respect to their acute mechanisms of ROS generation and genotoxicity [36,136]. However, the dose–response relationship between chronic, low-level dietary exposure to these compounds and the cumulative oxidative tissue damage that underpins the chronic diseases described in Section 4 remains poorly defined in both humans and companion animals. The mechanistic studies reviewed in this paper have predominantly employed acute, high-dose exposures in rodent models that do not recapitulate the lifelong, low-level dietary oxidant burden characteristic of habitual ultra-processed food consumption in humans [31] or exclusive commercial pet-food consumption in companion animals [46].

This gap is particularly consequential for companion animals, who—as noted in Section 5.1—may consume a single commercial diet formulation exclusively across their entire lifespan, a dietary monotony that has no human equivalent and that creates conditions for cumulative pro-oxidant burden amplification that are currently unquantified [51]. The 14-year controlled feeding study in Labrador retrievers, which demonstrated that dietary energy restriction extended lifespan by 1.8 years, with delayed onset of chronic diseases, illustrates both the feasibility and the scientific value of long-duration dietary intervention studies in companion animals [271], yet no equivalent study has systematically manipulated dietary pro-oxidant load while tracking tissue-level ROS biomarkers across the lifespan. Longitudinal cohort studies measuring urinary 8-OHdG, plasma F2-isoprostanes, erythrocyte antioxidant enzyme activities, and lipid peroxidation markers from early adulthood through senescence—in both humans and companion animals maintained on defined dietary regimens—are needed to establish species-appropriate dose–response thresholds relevant to disease prevention.

### 7.2. Lack of Validated Biomarker Frameworks for Dietary Pro-Oxidant Exposure in Companion Animals

Section 5.3 identified substantial interspecies differences in the toxicokinetic processing of dietary pro-oxidants: cats are deficient in hepatic glucuronyl transferase activity and possess reduced sulfotransferase capacity, severely impairing phase II conjugation of activated HCA metabolites and phenolic compounds and prolonging tissue exposure to reactive intermediates generated by a CYP system that remains functional [297]. Canine *CYP1A2* polymorphism creates marked inter-individual variability in HCA bioactivation among dogs, with affected individuals exhibiting substantially altered pharmacokinetics of CYP1A2-metabolized compounds [297]. Despite these well-characterized pharmacogenomic vulnerabilities, no validated, species-specific biomarker panel exists for non-invasive monitoring of dietary pro-oxidant exposure in clinical canine or feline populations.

The hemoglobin adducts of acrylamide and glycidamide—widely used as exposure biomarkers in human dietary risk assessment—have been characterized in humans but not systematically applied in companion-animal subjects under dietary exposure conditions representative of commercial pet-food consumption [280,291]. Similarly, urinary HCA metabolite profiling, which is a standard epidemiological tool in human cancer research, has been documented to detect HCA metabolites following processed diet consumption in dogs but has not been validated for routine application in companion-animal clinical or nutritional studies [176,191]. The development and validation of a companion-animal dietary oxidant exposure biomarker panel–integrating hemoglobin adducts, urinary oxidative DNA damage markers (8-OHdG), plasma lipid peroxidation products (4-HNE, MDA, and F2-isoprostanes), and HCA metabolite profiles, calibrated to species-specific toxicokinetics—would provide the methodological infrastructure necessary to translate the mechanistic insights reviewed in Section 5.3 into clinical risk stratification tools.

### 7.3. Oxidative Deterioration of Pet-Food Antioxidant Systems During Post-Opening Storage: An Unquantified Consumer-Level Exposure Gap

Section 5.1 identified a clinically relevant nutritional vulnerability specific to companion animals: tocopherol-based antioxidant systems in commercial dry pet-food kibble, formulated to meet AAFCO minimum requirements at the time of manufacture, undergo progressive oxidative degradation during storage—particularly following bag opening and repeated atmospheric oxygen exposure—yet this depletion is not captured in regulatory compliance frameworks that assess antioxidant adequacy at the point of production [51]. The rendered animal-fat fractions incorporated into pet foods as palatability enhancers are particularly susceptible to lipid oxidation during manufacturing and post-opening storage, and companion animals may receive a higher dietary burden of secondary lipid oxidation products (4-HNE, MDA, and lipid hydroperoxides) per kilogram of body weight than humans consuming typical mixed diets [49]. As a consequence, the actual dietary antioxidant status of companion animals consuming kibble under typical household storage conditions may be substantially lower than that implied by label declarations or AAFCO compliance data, and secondary lipid oxidation products may accumulate to levels that exceed those present at manufacture—a scenario with direct implications for the chronic oxidative stress burden documented in Section 4.4.

The chronic consumption of processed pet foods with inadequate antioxidant supplementation—particularly those containing rendered animal fats susceptible to lipid oxidation during storage—may contribute to the oxidative burden underlying obesity-associated comorbidities and other chronic diseases in companion animals, though controlled dietary intervention studies establishing this causal chain in vivo remain limited [254,272]. Systematic analytical studies measuring peroxide value, aldehyde content (4-HNE, MDA, and acrolein), and tocopherol concentration in commercially available dry pet foods at multiple post-opening timepoints under ecologically relevant storage conditions are needed to characterize consumer-level dietary oxidant exposure in companion animals and to provide the empirical basis for evidence-based revision of antioxidant adequacy standards in companion-animal nutrition.

### 7.4. Gut Microbiome as an Uncharacterized Mediator of Diet-Induced Oxidative Stress in Companion Animals

Section 1.3 identified gut microbiota remodeling as one of the mechanistic pathways through which ultra-processed food consumption promotes oxidative stress, operating through disruption of intestinal-barrier integrity, endotoxemia-driven systemic oxidative signaling, and the biotransformation of dietary pro-oxidants by microbial enzymes [40,41]. Section 4.5.2 further documented that commercial pet food additives—including emulsifiers carboxymethylcellulose and polysorbate 80—disrupt intestinal-barrier integrity, increase mucosal permeability to luminal antigens and LPS, and promote low-grade mucosal inflammation and ROS generation in dogs [264,266]. However, the specific mechanistic chain linking commercial pet-food composition to measurable alterations in companion-animal intestinal microbial community structure and from those alterations to quantifiable changes in host redox status has not been established at the molecular level.

This gap is particularly significant because the dietary monotony characteristic of exclusive commercial pet-food consumption—with its constrained substrate diversity and fixed additive profile—likely imposes a structurally distinct pattern of microbiome remodeling compared with the more variable dietary exposures of humans, potentially amplifying dysbiosis-driven oxidative burden in ways that are not predictable from human microbiome research. The concurrent depletion of dietary antioxidants that further impairs antioxidant defenses and amplifies susceptibility to oxidative damage [42] adds a further dimension to this microbiome–redox interaction that warrants systematic investigation. Gnotobiotic companion-animal models colonized with defined, species-representative microbial communities, combined with metagenomic profiling and redox-targeted metabolomics, would provide a controlled experimental platform for establishing the causal chain from dietary composition to microbiome composition and host redox state in dogs and cats.

### 7.5. Combinatorial Pro-Oxidant Interactions Among Co-Occurring Processing-Induced Contaminants

Section 3 documented that the five major mechanistic pathways through which PICs generate intracellular ROS—receptor-mediated NADPH oxidase activation, CYP-dependent redox cycling, mitochondrial ETC impairment, lipid peroxidation cascade propagation, and antioxidant depletion—operate in a mutually reinforcing manner and noted that the co-occurrence of multiple PICs in thermally processed diets creates a combinatorial oxidant burden whose magnitude likely exceeds simple additivity [12,136]. The experimental literature on individual PICs—acrylamide [280], HCAs [191,233], N-nitroso compounds [138], and lipid oxidation products [36]—has predominantly examined their pro-oxidant effects in isolation, at concentrations often exceeding those encountered in single-meal dietary exposure, and in single-compound experimental systems that preclude assessment of pathway convergence and antioxidant-system saturation under realistic dietary conditions.

The translational limitation of this single-compound experimental paradigm is most evident in the context of companion-animal nutrition, where the fixed commercial diet formulation simultaneously delivers the full PIC spectrum—Maillard reaction-derived AGEs, acrylamide, HCAs, PAHs, lipid peroxidation products, and preservative-associated oxidants—in every meal across the animal’s lifespan [46,49,176]. As demonstrated in the context of cancer and neurodegeneration across species, these multiple oxidant pathways converge on shared downstream targets, including NF-κB, Nrf2/ARE, and AP-1 signaling; redox-sensitive DNA repair enzymes; and mitochondrial ETC integrity [12,230]. Mixture toxicology approaches, including isobolographic analysis of pairwise and multi-compound PIC combinations and pathway-specific ROS flux measurements in organotypic cell systems exposed to authentic processed food or pet food extracts, would provide a quantitative basis for assessing whether the chronic disease burden documented in companion animals is attributable to individual PIC constituents or to synergistic interactions among them. These studies would simultaneously advance mechanistic understanding of real-world dietary oxidant risk and inform evidence-based reformulation strategies for both processed human foods and commercial pet-food products.

Collectively, these five knowledge gaps reflect a common underlying limitation: the mechanisms of dietary ROS-mediated pathology are substantially better characterized at the level of individual compounds and acute exposures than at the level of chronic, mixed, real-world dietary exposures in species with the metabolic and physiological heterogeneity that determines actual disease risk. As noted throughout this review, processing-induced toxic compounds and their ROS-mediated mechanisms represent an important but often under-recognized contributor to the initiation and progression of ROS-associated diseases across both humans and companion animals [136]. Addressing these gaps will require methodological advances in long-term dietary biomarker measurement, consumer-level food oxidation monitoring, species-specific toxicokinetic characterization, microbiome–redox interaction modeling, and mixture toxicology—advances most efficiently achieved through integrated cross-disciplinary collaboration between human nutrition science, veterinary medicine, food chemistry, and microbiology.

## 8. Literature Search and Selection Methodology

A comprehensive literature search was conducted to identify relevant studies on dietary ROS, oxidative stress, food- and feed-derived pro-oxidant compounds, and their associations with disease in humans and companion animals. The literature search was performed across multiple scientific databases, including PubMed, Google Scholar, Web of Science, and Scopus.

The search strategy was developed using keywords corresponding to the major topics covered in this review. The principal search terms included “reactive oxygen species”, “oxidative stress”, “humans”, “companion animals”, “dogs”, “cats”, “ROS-generating compounds in unprocessed foods”, “unprocessed foods”, “ROS-generating compounds in processed foods”, “processed foods”, “pro-oxidant compounds”, “metabolic diseases”, “neurodegenerative diseases”, “cancer”, “atherosclerosis”, and “veterinary diseases”. These terms were combined according to the major themes of the review, including dietary or feed exposure, ROS and oxidative stress, associated diseases, and target species. Boolean operators, including “AND” and “OR” were used to adjust the scope and relevance of the search results. Representative search combinations included “processed foods” AND “reactive oxygen species” AND “oxidative stress”.

The literature search covered publications available up to 10 July 2026, with no lower date restriction, and was restricted to English-language publications. Publications were primarily included when they were published in academic journals indexed in the Journal Citation Reports (JCR) and were relevant to the scope of this review. Publications from journals not indexed in the JCR were excluded. Non-academic or unsupported sources, including blog articles without identifiable scholarly sources, were also excluded.

## 9. Conclusions

Collectively, the evidence reviewed here establishes that dietary and feed-derived ROS and pro-oxidant compounds constitute a converging, modifiable driver of chronic disease in both humans and companion animals. Within the scope of this review, unprocessed foods and analogous thermal processing of human foods and commercial pet feeds generate a qualitatively similar spectrum of pro-oxidant toxicants that act through shared ROS-generating mechanisms, including receptor-mediated signaling, cytochrome P450 activation, mitochondrial dysfunction, lipid peroxidation, and progressive antioxidant depletion. This convergence is reflected epidemiologically in the parallel prevalence of metabolic disease, CKD, IBD, and cancer across species sharing a common domestic dietary environment, although the extent and manifestation of these diseases may vary among species because of species-specific toxicokinetic and lipoprotein–metabolic differences. Notably, dietary antioxidant compounds, themselves, can shift from protective antioxidant to ROS-generating pro-oxidant activity in a dose- and context-dependent manner, indicating that their intake and supplementation require careful, evidence-based dosing rather than indiscriminate use. These findings carry direct translational and practical implications. Companion animals, owing to their shared domestic exposures and compressed disease timelines, represent an underutilized naturalistic model for investigating diet-induced oxidative pathology and testing preventive dietary strategies applicable to both veterinary and human medicine. Future research should prioritize standardized, species-specific dose–response relationships and biomarker frameworks, together with the assessment of combinatorial pro-oxidant exposures that better reflect real-world dietary patterns. Addressing these key gaps will be important for the translation of mechanistic insight into evidence-based dietary interventions aimed at reducing the shared burden of oxidative stress-associated disease in humans and their companion animals.

## Figures and Tables

**Table 3:** Comparative patterns of oxidative stress-associated diseases across humans and companion animals (Section 5.4).

Disease/Condition	Pattern	Key Comparative Finding	Section Reference
Obesity-related metabolic disease	Parallel	Shared mechanisms—energy-dense processed food exposure, elevated NEFA-driven mitochondrial ROS, TNF-α-mediated IRS-1 serine phosphorylation, reduced adiponectin signaling, and systemic low-grade inflammation—drive comparable disease across humans, dogs, and cats; a 14-year Labrador retriever feeding study demonstrated the lifespan benefit of weight management.	Section 4.5.3
Chronic kidney disease	Parallel	Hyperfiltration-induced oxidative injury; qualitatively similar biomarker profiles; and shared therapeutic responsiveness to dietary protein restriction, phosphorus restriction, and omega-3 fatty acid supplementation characterize CKD progression in both humans and dogs.	Section 4.5.1
Atherosclerosis	Divergence	Common in humans but remarkably rare in companion animals—particularly cats, whose lipoprotein metabolism (absence of Cholesteryl Ester Transfer Protein (CETP)) limits atherogenic small, dense LDL formation—despite comparable dietary pro-oxidant exposure and obesity rates.	Section 4.4
Neurodegenerative disease (Alzheimer’s/CDS)	Divergence	Alzheimer’s disease, with its hallmark amyloid-β plaques and tau neurofibrillary tangles, does not occur naturally in dogs or cats; aged dogs instead develop a cognitive dysfunction syndrome (CDS) featuring amyloid-β accumulation and oxidative neuronal injury—but without the tau pathology characteristic of human AD—that parallels only early-stage human dementia.	Section 4.2.1
Cancer	Convergence	Shared ROS-mediated DNA damage, epigenetic silencing, and oncogenic signaling pathways underlie dietary HCA- and aflatoxin B1-associated carcinogenesis in humans, companion animals, and livestock alike.	Section 4.3, Section 4.5.4 and Section 5.3

**Table 4:** Processing-induced toxic compounds in human foods and pet feeds and their ROS-related mechanisms.

Matrix	Representative Toxicants	Typical Processing Conditions	ROS-Related Mechanisms	Key References
Human Food	HCAs (Heterocyclic amines)	High-temperature cooking (grilling, frying, and roasting)	CYP450-mediated metabolic activation → ROS generation and DNA adduct formation	[295]
Human Food	PAHs (Polycyclic aromatic hydrocarbons)	Direct flame grilling and smoking	CYP1A1-mediated metabolism → increased ROS production and promotion of carcinogenesis	[296]
Human Food	AGEs (Advanced glycation end products)	Maillard reaction during high-temperature processing (baked goods and meats)	RAGE activation → NADPH oxidase-mediated ROS generation	[20]
Pet Feed	HCAs (Heterocyclic amines)	High-temperature extrusion and thermal processing	CYP450-mediated metabolic activation → ROS generation and DNA adduct formation	[176,191,297]
Pet Feed	AGEs (Advanced glycation end products)	Maillard reaction during extrusion and heat processing	RAGE activation → NADPH oxidase-mediated ROS generation	[36,49]

## Data Availability

No new data were created or analyzed in this study. Data sharing is not applicable to this article.

## Abbreviations

The following abbreviations are used in this manuscript:

3-MCPD	3-monochloropropane-1,2-diol
4-HHE	4-hydroxy-2-hexenal
4-HNE	4-hydroxy-2-nonenal
8-OHdG	8-hydroxy-2′-deoxyguanosine
AD	Alzheimer’s disease
AFB1	Aflatoxin B1
AGEs	Advanced glycation end-products
AhR	Aryl hydrocarbon receptor
Akt	Protein kinase B
AMPK	AMP-activated protein kinase
AP-1	Activator protein 1
ARE	Antioxidant response element
ATP	Adenosine triphosphate
Aβ	Amyloid beta
BaP	Benzo[a]pyrene
BHA	Butylated hydroxyanisole
BHT	Butylated hydroxytoluene
CAT	Catalase
CDS	Cognitive dysfunction syndrome
CKD	Chronic kidney disease
CML	Nε-carboxymethyllysine
CNS	Central nervous system
CoQ10	Coenzyme Q10
CRC	Colorectal cancer
CYP	Cytochrome P450
DHA	Docosahexaenoic acid
DNA	Deoxyribonucleic acid
EGCG	Epigallocatechin-3-gallate
EPA	Eicosapentaenoic acid
ER	Endoplasmic reticulum
ETC	Electron transport chain
GPx	Glutathione peroxidase
GPx4	Glutathione peroxidase 4
GSH	Reduced glutathione
GSSG	Oxidized glutathione
GST	Glutathione S-transferase
HAT	Hydrogen atom transfer
HCAs	Heterocyclic aromatic amines
HCC	Hepatocellular carcinoma
HO-1	Heme oxygenase-1
H_2_O_2_	Hydrogen peroxide
IARC	International Agency for Research on Cancer
IBD	Inflammatory bowel disease
IL-6	Interleukin-6
JNK	c-Jun N-terminal kinase
Keap1	Kelch-like ECH-associated protein 1
LDL	Low-density lipoprotein
LOPs	Lipid oxidation products
LPS	Lipopolysaccharide
MAM	Mitochondria-associated membrane
MDA	Malondialdehyde
MG-H1	Methylglyoxal-derived hydroimidazolone 1
MnSOD	Manganese superoxide dismutase
mTOR	Mechanistic target of rapamycin
NAFLD	Nonalcoholic fatty liver disease
NASH	Nonalcoholic steatohepatitis
NF-κB	Nuclear factor kappa B
NO	Nitric oxide
NOCs	N-nitroso compounds
Nox/NOX	NADPH oxidase
NQO1	NAD(P)H quinone oxidoreductase 1
Nrf2/NRF2	Nuclear factor erythroid 2-related factor 2
O_2_•^−^	Superoxide anion radical
oxLDL	Oxidized low-density lipoprotein
•OH	Hydroxyl radical
PAHs	Polycyclic aromatic hydrocarbons
PD	Parkinson’s disease
PGC-1α	Peroxisome proliferator-activated receptor gamma coactivator 1 α
PICs	Processing-induced contaminants
PUFAs	Polyunsaturated fatty acids
RAGE	Receptor for advanced glycation end-products
ROO•	Peroxyl radical
ROS	Reactive oxygen species
SCFA	Short-chain fatty acid
SET	Single electron transfer
SIRT1	Sirtuin 1
SOD	Superoxide dismutase
SPMs	Specialized pro-resolving mediators
T2DM	Type 2 diabetes mellitus
TBARS	Thiobarbituric acid-reactive substances
TBHQ	tert-butylhydroquinone
TNF-α	Tumor necrosis factor α
TRX2	Mitochondrial thioredoxin 2
TrxR	Thioredoxin reductase
UPFPs	Ultra-processed food products
UPR	Unfolded protein response
^1^O_2_	Singlet oxygen
